# Association of CTI and its obesity-related derivatives with incident depression among middle-aged and older adults across CKM stages 0-4: a nationwide prospective cohort study and external clinical validation

**DOI:** 10.3389/fendo.2026.1849662

**Published:** 2026-05-20

**Authors:** Rui Zhang, Bin Yang, Na-Na Pan, Yi An, Qing Yu

**Affiliations:** 1 Department of Cardiology, The Affiliated Hospital of Qingdao University, Qingdao, China; 2 Department of Endocrinology and Metabolism, The Affiliated Hospital of Qingdao University, Qingdao, China

**Keywords:** cardiovascular-kidney-metabolic syndrome, Chinese visceral adiposity index, C-reactive protein-triglycerides-glucose index, cumulative exposure, depression

## Abstract

**Background:**

Cardiovascular-kidney-metabolic (CKM) syndrome integrates metabolic, renal, and cardiovascular risks. While C-reactive protein-triglycerides-glucose (CTI)-related indices are associated with future depression risk, their link to incident depression in populations across CKM stages 0–4 remains unestablished.

**Methods:**

This prospective cohort included 3,130 depression-free participants across CKM stages 0–4 from the China Health and Retirement Longitudinal Study. Four machine learning algorithms determined the core covariates for multivariable adjustments. Receiver operating characteristic (ROC) curves, net reclassification improvement (NRI), and integrated discrimination improvement (IDI) assessed the predictive performance of CTI-related indices. Multivariable Cox models, restricted cubic splines, and K-means clustering evaluated associations of the optimal indicator’s baseline, cumulative, and trajectory exposures with incident depression. An independent clinical cohort (n=350) provided directional replication.

**Results:**

Over a 9-year median follow-up, 1,275 participants developed depression. The CTI-Chinese visceral adiposity index (CTI-CVAI) achieved the highest AUC (0.705), providing incremental predictive value (NRI = 0.126, IDI = 0.005; *P* < 0.001). The fully adjusted model revealed a decreased depression risk with higher baseline CTI-CVAI (HR per 1-SD=0.91, 95% CI: 0.85-0.96). A linear dose-response relationship was observed for both baseline and cumulative CTI-CVAI. Furthermore, individuals in the high-increasing trajectory group (HR = 0.81, 95% CI: 0.69-0.97) and the highest cumulative exposure tertile (HR = 0.87, 95% CI: 0.76-0.97) exhibited significantly lower depression risks. This inverse association was primarily prominent in participants aged <60 years (*P* for interaction=0.032). The external validation cohort replicated the moderate discriminatory power (AUC = 0.691) and the independent inverse association of CTI-CVAI with incident depressive symptoms (OR per 1-SD=0.92, *P* < 0.001).

**Conclusions:**

CTI-CVAI is an independent predictor with moderate discrimination for incident depression in populations across CKM stages 0-4. These findings support its potential as a candidate marker, suggesting moderate cardiometabolic and nutritional reserves may be associated with better mental health in middle-aged and older adults across CKM stages 0-4.

## Introduction

Cardiovascular-kidney-metabolic (CKM) syndrome, a framework recently conceptualized by the American Heart Association in 2023, integrates the pathophysiological interactions among metabolic disorders, chronic kidney disease, and cardiovascular diseases (1, 2). Individuals across the CKM spectrum frequently experience cardiometabolic and psychosocial risk clustering, including depressive symptoms (3, 4). The coexistence of depression and CKM components accelerates physical and cognitive decline, and significantly increases mortality (5, 6). Recent epidemiological evidence highlights a complex bidirectional relationship between cardiometabolic disorders and mental health, driven by shared pathways such as systemic inflammation, oxidative stress, and neuroendocrine dysregulation (7). Given this interconnected risk, identifying objective and accessible biomarkers to stratify incident depression risk in populations across CKM stages 0–4 prior to clinical manifestation has emerged as a clinical priority.

In populations spanning CKM stages 0-4, depression risk may arise from overlapping inflammatory, metabolic, renal, cardiovascular, and psychosocial pathways (8, 9). The C-reactive protein-triglycerides-glucose (CTI) index has recently been proposed as a composite indicator reflecting both chronic low-grade inflammation and IR (10, 11). Furthermore, integrating anthropometric measurements to form CTI’s obesity-related derivatives (e.g., CTI-CVAI, CTI-BMI, CTI-WC) can capture a broader spectrum of pathophysiological features, including visceral adiposity and body shape distribution (12, 13). While recent studies have linked depressive symptoms to the progression of CKM syndrome (14), the predictive value of the CTI and its obesity-related derivatives for incident depression remains to be fully evaluated. Furthermore, the relationship between cardiometabolic burden and late-life mental health is subject to ongoing debate (15). While severe metabolic dysfunction is traditionally viewed as detrimental, the “obesity paradox” or “nutritional reserve” hypothesis posits that moderate adiposity and cardiometabolic reserves may exert a neuroprotective buffering effect against psychosocial stress in older adults (16–18).

To address these knowledge gaps, this nationwide prospective cohort study utilized data from the China Health and Retirement Longitudinal Study (CHARLS). We aimed to systematically investigate the longitudinal associations of the CTI and its obesity-related derivatives with incident depression among middle-aged and older adults across CKM stages 0-4. Specifically, we applied integrated machine learning algorithms to identify the optimal multidimensional predictor, and evaluated the relationship between the optimal indicator’s baseline level, cumulative exposure, and dynamic evolving trajectories with the risk of incident depression. By dissecting these multidimensional relationships, this study seeks to provide a discriminative screening tool and offer epidemiological insights into the cardiometabolic-nutritional paradox in middle-aged and older adults across CKM stages 0-4, with core findings further corroborated by an independent real-world clinical cohort.

## Methods

### Study design and population

Data for this study were obtained from the CHARLS, an ongoing, nationally representative prospective cohort study covering Chinese adults aged 45 years and older. Detailed descriptions of the CHARLS study design and methodology have been published previously (19). The study protocol was approved by the Biomedical Ethics Review Committee of Peking University (IRB00001052-11015) and was conducted in accordance with the principles of the Declaration of Helsinki. All participants provided written informed consent prior to data collection.

**Figure 1:**
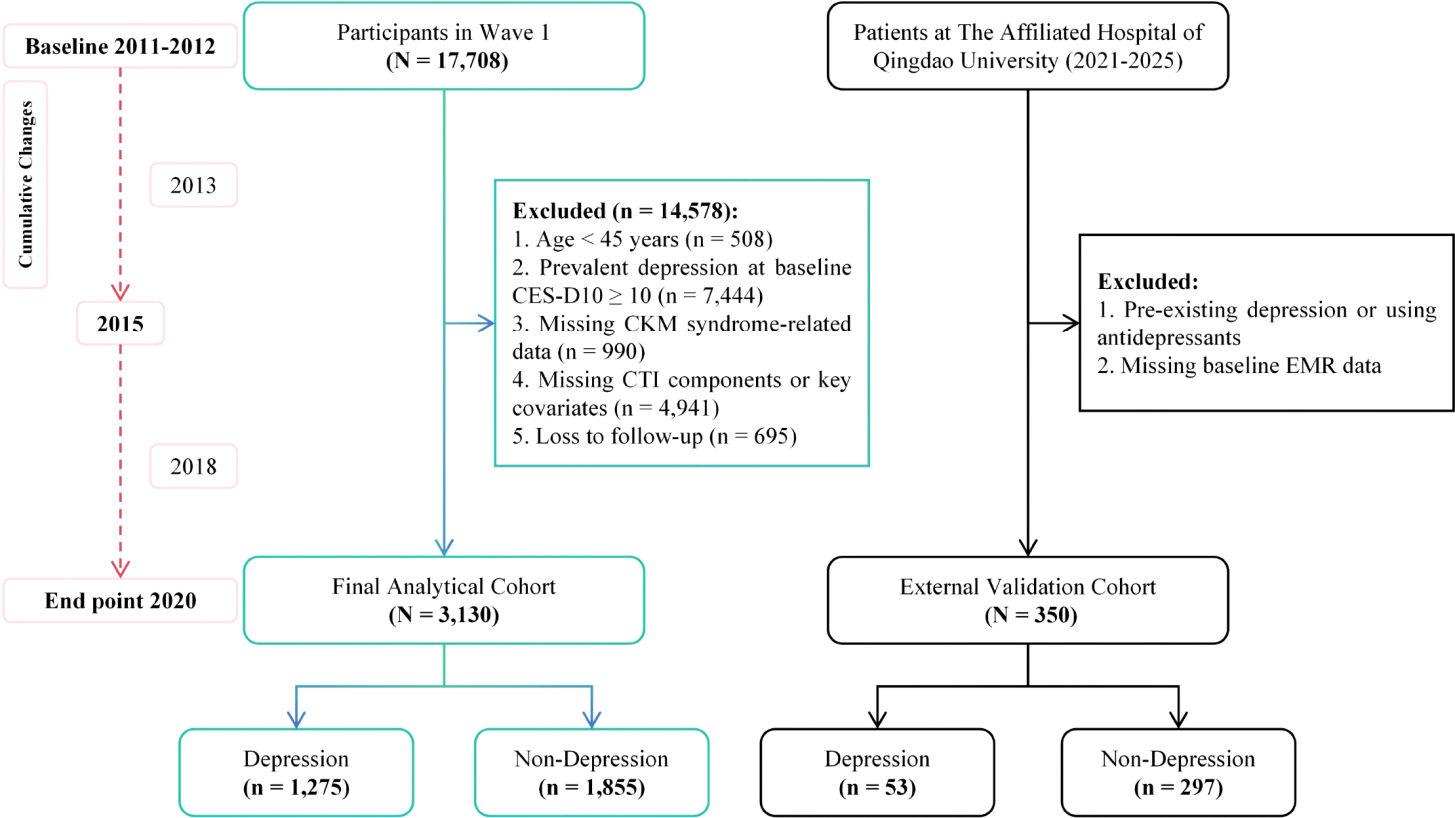
Flowchart of the study population selection.

The current study utilized baseline data from Wave 1 (2011-2012) and longitudinal data from subsequent follow-up waves up to Wave 5 (2020). As illustrated in **Figure 1**, a total of 17,708 participants were initially enrolled at baseline. Participants were sequentially excluded based on the following criteria: (1) aged < 45 years (n = 508); (2) prevalent depression at baseline, defined as a 10-item Center for Epidemiologic Studies Depression Scale (CES-D 10) score ≥ 10 (n = 7,444); (3) missing data related to CKM syndrome (n = 990); (4) missing components required for calculating the CTI-derived indices or essential covariates (n = 4,941); and (5) loss to follow-up during the subsequent waves (n = 695). Following these exclusions, a final cohort of 3,130 eligible participants was included in the present analysis.

### Definition and staging of CKM syndrome

In accordance with the 2023 American Heart Association (AHA) Presidential Advisory (20), CKM syndrome staging (Stages 0-4) was systematically determined based on the presence of metabolic risk factors, chronic kidney disease (CKD), and subclinical or clinical cardiovascular disease (CVD). Specifically, the stages were classified as follows: stage 0 indicates the absence of CKM risk factors; stage 1 is characterized by excess or dysfunctional adiposity (e.g., overweight/obesity, abdominal obesity) or prediabetes; stage 2 includes metabolic risk factors (hypertension, diabetes mellitus, hypertriglyceridemia, or metabolic syndrome) or moderate-to-high risk CKD; stage 3 represents subclinical CVD (defined as a high predicted 10-year CVD risk or very high-risk CKD); and stage 4 represents established clinical CVD (e.g., coronary heart disease, heart failure, or stroke). In the present study, to comprehensively evaluate the longitudinal impact of cardiometabolic burden on late-life mental health, individuals across CKM stages 0–4 at baseline were included in the primary analytical cohort. This approach ensures the cohort represents a general population of middle-aged and older adults, rather than strictly those with established CKM syndrome.

### Assessment of CTI-related indices and cumulative exposure

Baseline fasting venous blood samples and standardized anthropometric measurements were utilized to calculate the CTI index and its obesity-related derivatives. CTI serves as a composite indicator reflecting both systemic inflammation and insulin resistance (21). To comprehensively evaluate metabolic and visceral fat burden, several fundamental anthropometric and obesity-related indices were evaluated, including body mass index (BMI), waist circumference-to-height ratio (WHtR), body roundness index (BRI), weight-adjusted waist index (WWI), a body shape index (ABSI), visceral adiposity index (VAI), and the Chinese visceral adiposity index (CVAI). The precise mathematical formulas and definitions for CTI and these corresponding anthropometric indices are detailed in **Supplementary Table 1**. Subsequently, the comprehensive CTI-derived indices were constructed by multiplying the baseline CTI value by the respective anthropometric measurements: CTI-BMI, CTI-WC, CTI-WHtR, CTI-BRI, CTI-WWI, CTI-VAI, CTI-CVAI, and CTI-ABSI.

To capture the long-term burden of metabolic dysfunction, cumulative exposure to CTI-CVAI (CuCTI-CVAI) was calculated using a trapezoidal area-under-the-curve approach based on repeated measurements. The CuCTI-CVAI was mathematically defined as (22): CuCTI-CVAI=(CTI-CVAI_2012_ + CTI-CVAI_2015_)/2 × time (2015-2012). This cumulative metric effectively integrates both the magnitude and duration of the exposure over the longitudinal tracking period. Furthermore, to elucidate dynamic temporal patterns, a k-means clustering algorithm based on Euclidean distance was applied to CTI-CVAI measurements across the initial survey waves. The optimal number of clusters (K = 3) was robustly determined using the elbow method, effectively categorizing participants into distinct longitudinal trajectories.

### Ascertainment of incident depression and covariates

The primary outcome was incident depression, ascertained during the follow-up interviews using the CES-D 10. Incident depression was defined as a CES-D 10 score ≥ 10 at any follow-up visit among participants who were free of depression at baseline. Baseline covariates were systematically collected during the initial survey wave and grouped into specific clinical and sociodemographic domains for comprehensive analysis. Demographic characteristics included age, sex, marital status, educational level, and household registration type (Hukou). Lifestyle habits comprised smoking status, alcohol consumption, and nocturnal sleep time. Psychosocial and functional well-being were assessed comprehensively: physical function was evaluated via the Activities of Daily Living (ADL) scale; global cognitive function was determined by assessing episodic memory and mental state; self-rated health (SRH) was classified into five descriptive categories (excellent, very good, good, fair, and poor); and the level of social isolation was categorized into five tiers indicating isolation severity (none, mild, moderate, high, and extreme). Furthermore, anthropometric and physiological measurements consisted of height, weight, waist circumference (WC), body mass index (BMI), systolic blood pressure (SBP), and diastolic blood pressure (DBP). Laboratory assessments involved the quantitative analysis of fasting venous blood samples, capturing fasting plasma glucose (FPG), glycated hemoglobin (HbA1c), triglycerides (TG), total cholesterol (TC), high-density lipoprotein cholesterol (HDL-C), low-density lipoprotein cholesterol (LDL-C), C-reactive protein (CRP), blood urea nitrogen (BUN), serum creatinine (Cr), and uric acid (UA). Finally, underlying cardiometabolic comorbidities—specifically hypertension, diabetes, and dyslipidemia—along with the concurrent use of corresponding pharmacological treatments (antihypertensive, antidiabetic, and lipid-lowering drugs) were systematically recorded.

### External validation dataset

To provide directional replication of the main findings, we used an independent retrospective clinical cohort. We retrospectively included patients aged ≥ 45 years across CKM stages 0–4 who underwent routine clinical examinations at The Affiliated Hospital of Qingdao University between January 2021 and January 2025. Similar inclusion and exclusion criteria were applied to ensure comparability with the primary CHARLS cohort. Specifically, participants with pre-existing depression or those taking antidepressant medications at baseline were strictly excluded. Ultimately, the validation dataset comprised 350 participants. Ethical approval for this retrospective validation study was obtained from the Institutional Review Board of The Affiliated Hospital of Qingdao University, and the requirement for written informed consent was waived.

### Covariates and outcome assessment in the validation cohort

Baseline parameters required for calculating the CTI-CVAI were extracted from the electronic medical records (EMR) system. In this validation cohort, incident depression was primarily assessed using the 9-item Patient Health Questionnaire (PHQ-9). In accordance with established literature and routine clinical practice, a PHQ-9 score of ≥ 10 was utilized as the optimal cut-off to define positive incident depression. Furthermore, all corresponding baseline covariates aligned with the Model 3 (machine-learning-adjusted model) in our primary analysis—including age, sex, hukou, drinking history, social isolation, self-rated health, cognitive score, and mental state—were extracted from clinical records and EMRs. Statistical analyses, including ROC curve generation and multivariable logistic regression, followed the identical analytical framework as the primary study to verify the independent predictive value of CTI-CVAI.

### Statistical analysis

Continuous variables were presented as medians with interquartile ranges (IQRs) and compared using the Wilcoxon rank-sum test. Categorical variables were expressed as counts with percentages and compared using the chi-square test. Participants with missing data for any of the required covariates were excluded, and a complete-case analysis approach was adopted. To address potential confounding and avoid high-dimensional data overfitting, a machine learning-based feature selection pipeline was implemented. Specifically, we utilized the Boruta algorithm to rank variable importance, Recursive Feature Elimination (RFE) to select the optimal subset, Least Absolute Shrinkage and Selection Operator (LASSO) regression to minimize 10-fold cross-validation error, and a Genetic Algorithm (GA) to optimize fitness scores. The consensus features identified across the intersection of these four algorithms were selected as core covariates for the subsequent multivariable adjustments. Prior to multivariable adjustments, potential multicollinearity among the selected core covariates was evaluated using the Variance Inflation Factor (VIF), with all VIF values strictly < 5, indicating no severe collinearity. The predictive performance of CTI and its obesity-related derivatives for incident depression was evaluated by constructing both conventional and time-dependent receiver operating characteristic (ROC) curves, with the corresponding area under the curve (AUC) calculated to assess discriminatory ability. To evaluate the longitudinal stability of predictive accuracy, dynamic AUC trends were further analyzed at specified follow-up time points (years 5, 7, and 9). The DeLong test was applied to statistically compare AUCs across different indices. Furthermore, the incremental predictive value of the optimal index (CTI-CVAI) beyond the baseline clinical model was quantified by calculating the continuous Net Reclassification Improvement (NRI) and Integrated Discrimination Improvement (IDI). To investigate longitudinal associations, the baseline levels, cumulative exposures, and dynamic trajectory clusters of CTI-CVAI were evaluated. For longitudinal analyses involving cumulative exposure and dynamic trajectories (which utilized data from Wave 2 and Wave 3), the follow-up period for incident depression was redefined to start after the exposure assessment window (i.e., post-2015). Therefore, participants who developed depression, died, or were lost to follow-up prior to the 2015 landmark were inherently excluded from the final analytical cohort to prevent immortal time bias and reverse causality. Kaplan-Meier survival curves were generated to compare cumulative depression incidence rates across strata, with differences assessed using log-rank tests. Multivariable Cox proportional hazards models were constructed to estimate hazard ratios (HRs) and 95% confidence intervals (CIs). The initial core covariates identified by the machine learning intersection were adjusted in Model 3. To rigorously address potential residual confounding based on prior clinical knowledge and causal structures, we further constructed a clinically-driven fully adjusted model (Model 4). Independent of the algorithmic selection, Model 4 incorporated clinically relevant confounders, including education, marital status, nocturnal sleep time, activities of daily living, baseline chronic comorbidities (hypertension, diabetes, and dyslipidemia), and corresponding medication use (antidiabetic, lipid-lowering, and antihypertensive drugs). The proportional hazards (PH) assumption was rigorously verified for all variables using Schoenfeld residuals, and no significant violations were observed. Additionally, restricted cubic spline (RCS) regression with designated knots was applied to evaluate potential non-linear dose-response relationships between CTI-CVAI (including baseline and cumulative exposures) and incident depression. Finally, stratified subgroup analyses and multiplicative interaction tests were performed to assess the robustness of the associations across diverse clinical profiles and to identify potential effect modifiers. For the external validation study, descriptive statistics and regression analyses followed the identical analytical framework as the primary CHARLS study. ROC curves were generated to evaluate the discriminatory power of baseline CTI-CVAI for incident depression in the clinical setting. Subsequently, multivariable regression models, fully adjusted for all available Model 3 covariates, were constructed to verify the independent predictive value of CTI-CVAI. Due to the inherent limitations of retrospective electronic medical records in accurately capturing precise time-to-event data across all clinical visits, multivariable logistic regression was pragmatically employed in the validation cohort to assess the independent association, reporting Odds Ratios (ORs) rather than Hazard Ratios (HRs). All statistical analyses were conducted using *R* software (version 4.5.1). Statistical significance was defined as a two-tailed *P*-value < 0.05.

## Results

### Baseline characteristics and machine learning-based feature selection

**Figure 2:**
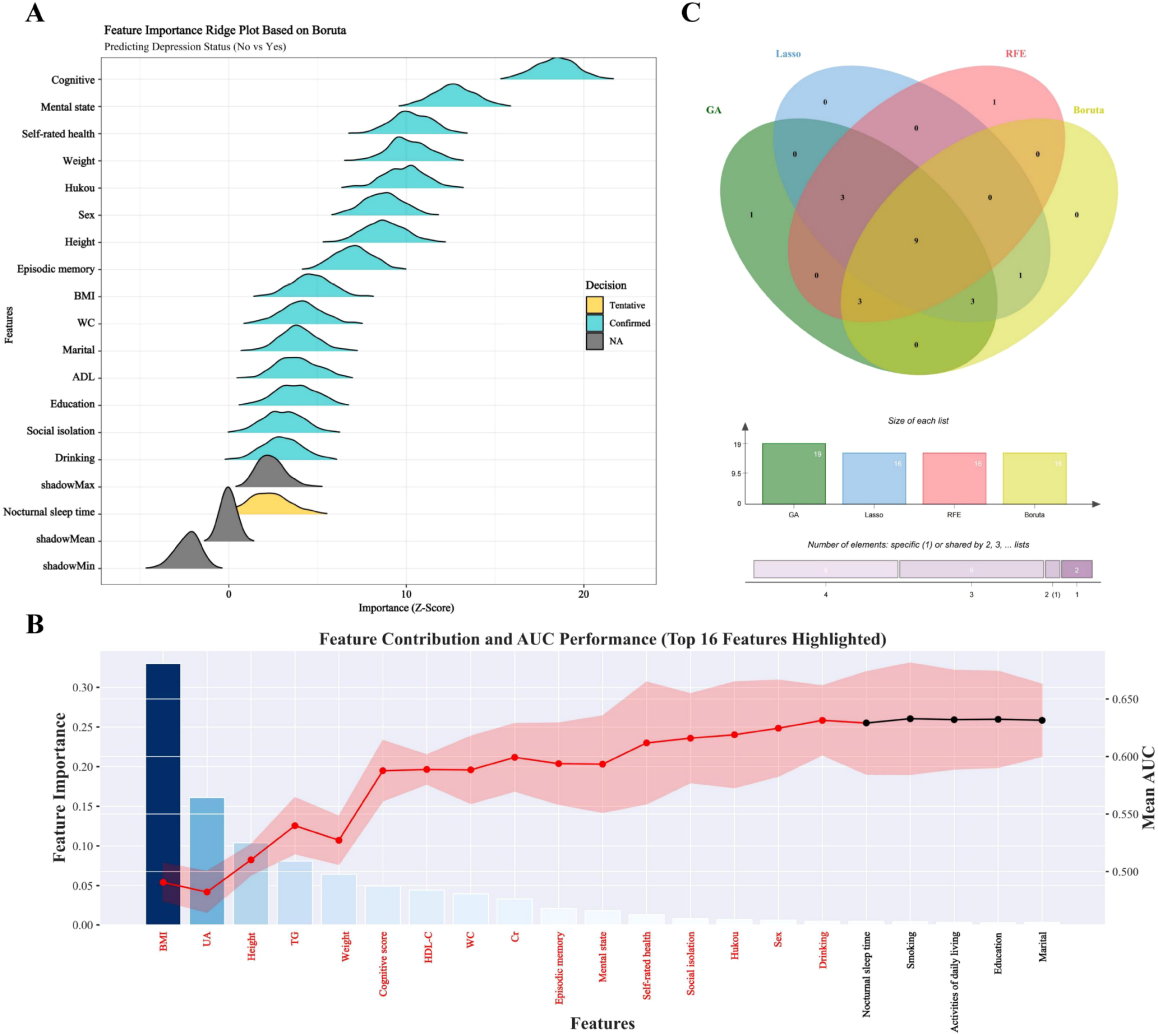
Identification of core covariates using an integrated machine learning-based feature selection pipeline [**(A)** Feature importance ridge plot based on Boruta; **(B)** Recursive feature elimination (RFE) for feature contribution screening; **(C)** Venn diagram illustrating the intersection of the core covariates selected by the four algorithms].

Baseline characteristics of the 3,130 participants, categorized by incident depression status, are summarized in **Supplementary Table 2**. During the follow-up period, 1,275 participants (40.7%) experienced incident depression. Compared with the non-depression group, individuals who developed depression were more likely to be female and unmarried, and had a lower proportion of rural Hukou. They also exhibited significantly lower mean values for weight, height, waist circumference, BMI, serum creatinine, uric acid, triglycerides, and high-density lipoprotein cholesterol, alongside poorer cognitive and mental state scores (all *P* < 0.05). Notably, baseline levels of CTI, CTI-BMI, CTI-WC, and CTI-CVAI were significantly lower in the incident depression group. Variables included in the final analytical models were selected through a systematic screening process (**Figure 2**, **Supplementary Figure 1**). The intersection of features prioritized by four complementary machine learning approaches—the Boruta algorithm, RFE, LASSO, and GA—yielded an initial set of 16 candidate variables. After integration of clinical relevance and removing collinear predictors, 8 core covariates were ultimately included in the final adjusted models: age, sex, hukou, drinking, social isolation, self-rated health, cognitive score, and mental state.

To assess the potential impact of sample attrition, we compared the baseline characteristics of the final analytical cohort (n = 3,130) with the eligible participants who were excluded due to missing data or loss to follow-up (n = 6,626) (**Supplementary Table 3**). While the two groups were comparable in age, self-rated health, and general anthropometric or laboratory measurements (e.g., BMI, blood pressure, fasting plasma glucose), systematic differences were observed in specific clinical and psychosocial domains. Compared to the excluded population, the included participants had a lower proportion of rural residents (35.59% vs. 49.19%, *P* < 0.001), a lower baseline prevalence of hypertension (23.19% vs. 25.28%, *P* = 0.001) and diabetes (4.98% vs. 6.08%, *P* < 0.001), and different distributions in mental state scores, activities of daily living, and social isolation. This indicates underlying differences in comorbidity burdens and sociodemographic backgrounds.

### Predictive performance of CTI-related indices

**Figure 3:**
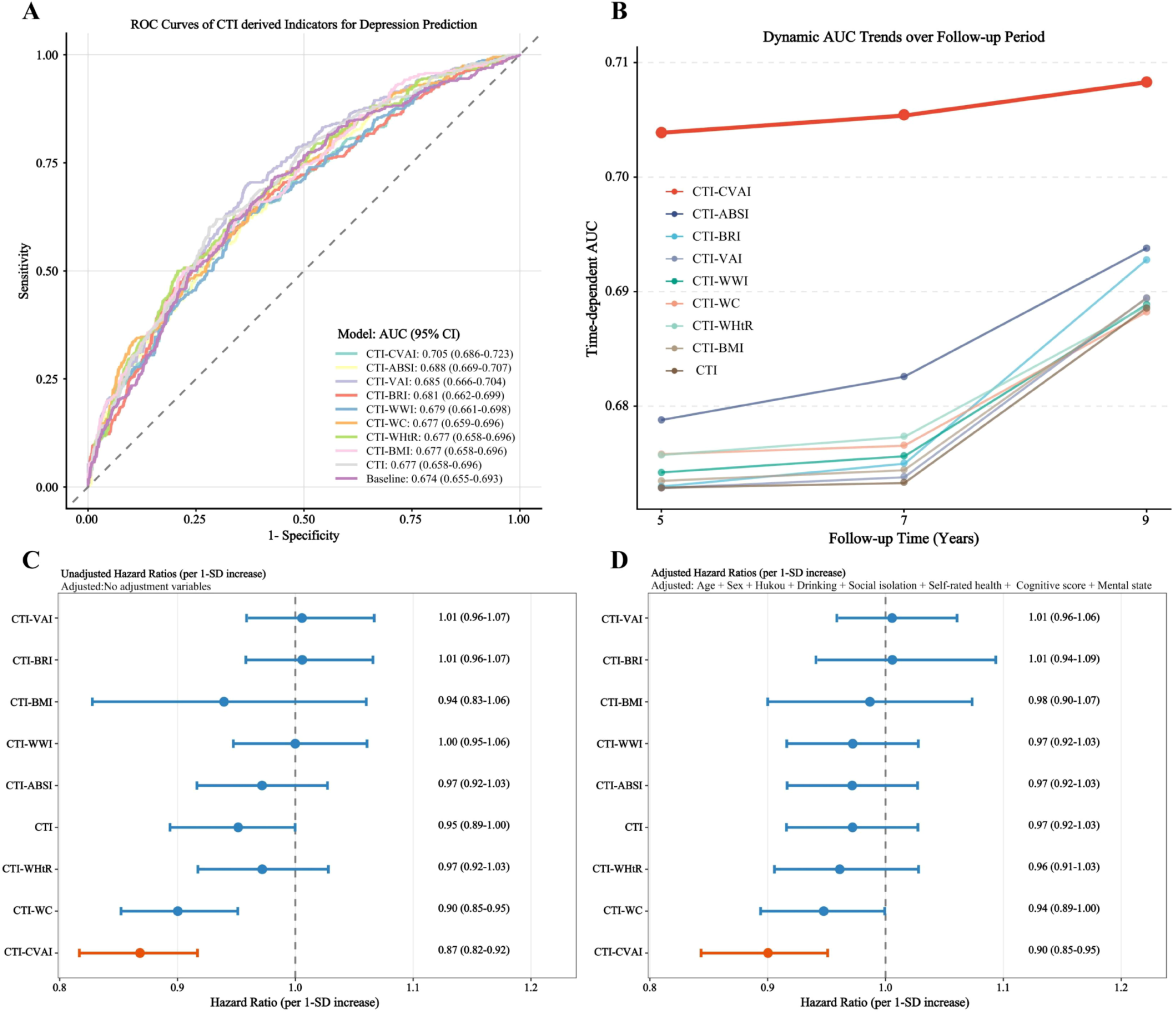
Predictive performance and risk associations of CTI and its obesity-related derivatives for incident depression. [**(A)** Conventional ROC curves comparing the predictive capabilities of different indices; **(B)** Dynamic trends of time-dependent AUCs for the evaluated indices over the 9-year follow-up period; **(C)** Unadjusted hazard ratios for incident depression per 1-SD increment; **(D)** Fully adjusted hazard ratios for incident depression per 1-SD increment].

To identify the optimal predictor for incident depression, the discriminatory performance of CTI and its eight derived indices was evaluated using conventional and time-dependent ROC curves. Among all evaluated metrics, CTI-CVAI demonstrated the highest area under the curve (AUC) of 0.705 (95% CI: 0.686-0.723), outperforming the baseline clinical model (AUC: 0.674) and other derivatives such as CTI-ABSI (0.688) and CTI-VAI (0.685) (**Figure 3A**). Furthermore, dynamic AUC trend analyses over the 9-year follow-up period revealed that CTI-CVAI consistently maintained the highest predictive accuracy across various time points (years 5, 7, and 9) compared to all other candidates (**Figure 3B**). Additionally, CTI-CVAI exhibited the strongest inverse association with incident depression per 1-SD increment in both unadjusted (HR = 0.87, 95% CI: 0.82-0.92) and fully adjusted models (HR = 0.90, 95% CI: 0.85-0.95) (**Figures 3C, D**).

The incremental predictive utility of CTI-CVAI was further quantified using reclassification metrics (**Supplementary Table 4**). Incorporating baseline CTI-CVAI into the reference clinical model significantly improved risk stratification, yielding a NRI of 0.126 (95% CI: 0.072-0.181; *P* < 0.001) and an IDI of 0.005 (95% CI: 0.003-0.007; *P* < 0.001). Consequently, CTI-CVAI was selected as the primary exposure variable for all subsequent longitudinal analyses. However, the small IDI value suggests that the practical incremental gain over the baseline clinical model may be limited.

### Characteristics according to CTI-CVAI tertiles

Baseline characteristics of the 3,130 participants stratified by CTI-CVAI tertiles are presented in **Table 1**. The incidence of depression decreased progressively across the tertiles, from 43.68% in Q1 to 41.04% in Q2, and 37.49% in Q3 (*P* = 0.015). Compared with Q1, individuals in the highest tertile (Q3) were older and had higher mean values for weight, waist circumference, BMI, systolic and diastolic blood pressure, fasting plasma glucose, HbA1c, uric acid, total cholesterol, triglycerides, LDL-C, and CRP. In contrast, participants in Q3 had significantly lower HDL-C levels. The Q3 group also comprised a higher proportion of females, rural residents, and individuals with advanced CKM stages, hypertension, diabetes, and dyslipidemia, whereas current smoking, drinking, and social isolation were less prevalent in this tertile.

**Table 1:** Baseline characteristics of the study population stratified by CTI-CVAI tertiles.

Variables	Q1 (n = 1044)	Q2 (n = 1043)	Q3 (n = 1043)	P
Depression, n(%)	456 (43.68)	428 (41.04)	391 (37.49)	0.015
Age, years	55.00 (49.00,61.00)	57.00 (50.00,62.00)	59.00 (54.00,64.00)	<0.001
SBP, mmHg	119.50 (109.25,131.50)	124.50 (113.00,139.50)	133.50 (121.00,146.50)	<0.001
DBP, mmHg	71.50 (64.25,79.50)	74.50 (67.50,82.50)	78.50 (70.50,86.50)	<0.001
Weight, kg	53.50 (48.40,58.40)	59.70 (53.85,65.00)	68.60 (62.05,75.80)	<0.001
Height, m	1.59 (1.53,1.65)	1.58 (1.52,1.65)	1.60 (1.53,1.66)	0.001
WC, cm	77.00 (72.80,80.02)	86.00 (82.00,89.00)	95.50 (91.00,100.00)	<0.001
BMI, kg/m2	21.11 (19.54,22.64)	23.58 (22.11,25.06)	26.76 (24.83,28.93)	<0.001
BUN, mg/dL	15.15 (12.54,18.32)	14.85 (12.52,17.65)	15.01 (12.70,17.42)	0.082
Cr, mg/dL	0.76 (0.66,0.87)	0.75 (0.64,0.87)	0.78 (0.68,0.90)	<0.001
HbA1c, %	5.10 (4.80,5.30)	5.10 (4.90,5.40)	5.20 (5.00,5.60)	<0.001
UA, mg/dL	4.08 (3.42,4.89)	4.14 (3.51,5.01)	4.66 (3.94,5.54)	<0.001
TC, mg/dL	182.09 (160.44,207.99)	190.21 (168.17,214.56)	197.55 (173.78,223.07)	<0.001
TG, mg/dL	74.34 (58.41,98.24)	106.20 (80.54,142.49)	163.73 (118.59,240.72)	<0.001
HDL-C, mg/dL	56.83 (48.71,67.65)	49.10 (41.75,57.22)	40.21 (33.63,47.55)	<0.001
LDL-C, mg/dL	107.86 (88.53,129.90)	116.75 (96.46,140.72)	117.53 (93.56,139.95)	<0.001
CRP, mg/L	0.65 (0.39,1.26)	0.94 (0.55,1.87)	1.51 (0.88,2.83)	<0.001
FPG, mg/dL	99.00 (91.98,106.60)	101.88 (94.77,110.97)	108.18 (99.18,122.76)	<0.001
Cognitive score	12.00 (9.00,14.50)	12.50 (9.00,14.50)	12.50 (9.00,15.00)	0.008
Episodic memory score	3.50 (2.00,5.00)	3.50 (2.00,5.00)	3.50 (2.00,5.00)	0.214
Mental state score	9.00 (6.00,10.00)	9.00 (6.00,10.00)	9.00 (7.00,11.00)	0.003
Male, n (%)	596 (57.09)	465 (44.58)	506 (48.51)	<0.001
Married, n (%)	930 (89.08)	934 (89.55)	942 (90.32)	0.646
Education, n (%)				0.259
< high school	931 (89.18)	937 (89.84)	914 (87.63)	
≥ high school	113 (10.82)	106 (10.16)	129 (12.37)	
Rural, n (%)	300 (28.74)	362 (34.71)	452 (43.34)	<0.001
Smoking, n (%)	483 (46.26)	353 (33.84)	408 (39.12)	<0.001
Drinking, n (%)	452 (43.30)	378 (36.24)	429 (41.13)	0.003
Hypertension, n (%)	105 (10.06)	221 (21.19)	400 (38.35)	<0.001
Diabetes, n (%)	19 (1.82)	46 (4.41)	91 (8.72)	<0.001
Dyslipidemia, n (%)	45 (4.31)	78 (7.48)	178 (17.07)	<0.001
Antidiabetic, n(%)	13 (1.25)	16 (1.53)	59 (5.66)	<0.001
Lipid-lowering, n (%)	15 (1.44)	43 (4.12)	96 (9.20)	<0.001
Antihypertensive, n (%)	61 (5.84)	144 (13.81)	312 (29.91)	<0.001
Activities of daily living, n (%)	75 (7.18)	84 (8.05)	124 (11.89)	<0.001
Nocturnal sleep time, n (%)				0.201
< 6 h	228 (21.84)	214 (20.52)	195 (18.70)	
≥ 6 h	816 (78.16)	829 (79.48)	848 (81.30)	
Social isolation, n (%)				0.031
None	417 (39.94)	460 (44.10)	493 (47.27)	
Mild	535 (51.25)	505 (48.42)	481 (46.12)	
Moderate	76 (7.28)	68 (6.52)	53 (5.08)	
High	13 (1.25)	10 (0.96)	14 (1.34)	
Extreme	3 (0.29)	0 (0.00)	2 (0.19)	
Self-rated health, n (%)				0.188
Excellent	19 (1.82)	19 (1.82)	15 (1.44)	
Very good	141 (13.51)	153 (14.67)	145 (13.90)	
Good	600 (57.47)	559 (53.60)	546 (52.35)	
Fair	215 (20.59)	228 (21.86)	235 (22.53)	
Poor	69 (6.61)	84 (8.05)	102 (9.78)	
CKM stage, n(%)				<0.001
Stage 0	198 (18.97)	30 (2.88)	0 (0.00)	
Stage 1	278 (26.63)	219 (21.00)	49 (4.70)	
Stage 2	290 (27.78)	454 (43.53)	412 (39.50)	
Stage 3	217 (20.79)	269 (25.79)	445 (42.67)	
Stage 4	61 (5.84)	71 (6.81)	137 (13.14)	

SBP, systolic blood pressure; DBP, diastolic blood pressure; WC, waist circumference; BMI, body mass index; BUN, blood urea nitrogen; Cr, serum creatinine; HbA1c, glycated hemoglobin; UA, uric acid; TC, total cholesterol; TG, triglycerides; HDL-C, high-density lipoprotein cholesterol; LDL-C, low-density lipoprotein cholesterol; CRP, C-reactive protein; FPG, fasting plasma glucose; CKM, cardiovascular kidney-metabolic syndrome.

### Dynamic trajectories of CTI-CVAI

**Figure 4:**
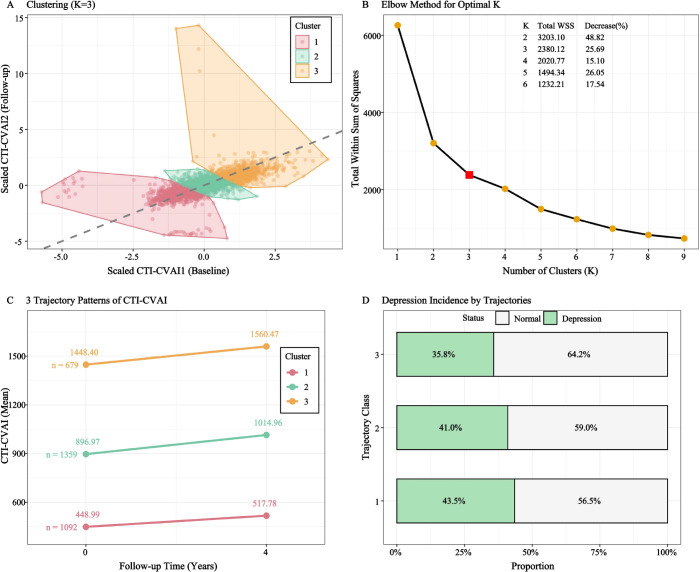
Identification and characteristics of the dynamic evolving trajectories of CTI-CVAI using K-means clustering. [**(A, C)** Longitudinal trajectory patterns; **(B)** Optimal cluster determination using the elbow method; **(D)** Incidence of depression across different trajectory clusters].

To capture the longitudinal evolution of the optimal predictor, K-means clustering analysis was applied to CTI-CVAI measurements across the initial two survey waves, identifying three distinct dynamic trajectories (**Figures 4A, C**). Cluster 1 (low-stable, n = 1,092) was characterized by maintaining a consistently low CTI-CVAI level, starting at 448.99 and slightly rising to 517.78. Cluster 2 (moderate-increasing, n = 1,359) exhibited an intermediate baseline level that increased from 896.97 to 1014.96. Cluster 3 (high-increasing, n = 679) started with the highest baseline burden and experienced a pronounced elevation from 1448.40 to 1560.47. The optimal number of clusters (K = 3) was robustly determined using the elbow method (**Figure 4B**).

Baseline characteristics of participants grouped by these three trajectory clusters are detailed in **Supplementary Table 5**. Consistent with the baseline tertile trends, individuals in the higher-burden trajectories (Clusters 2 and 3) generally exhibited older age, higher cardiometabolic derangements, and a greater prevalence of comorbidities. Notably, incident depression rates differed profoundly among the trajectory classes. The low-stable trajectory (Cluster 1) was associated with the highest depression incidence (43.5%), whereas the high-increasing trajectory (Cluster 3) had the lowest incidence (35.8%), indicating a graded relationship between longitudinal CTI-CVAI exposure and depression risk (**Figure 4D**).

### Associations of CTI-CVAI with incident depression

**Figure 5:**
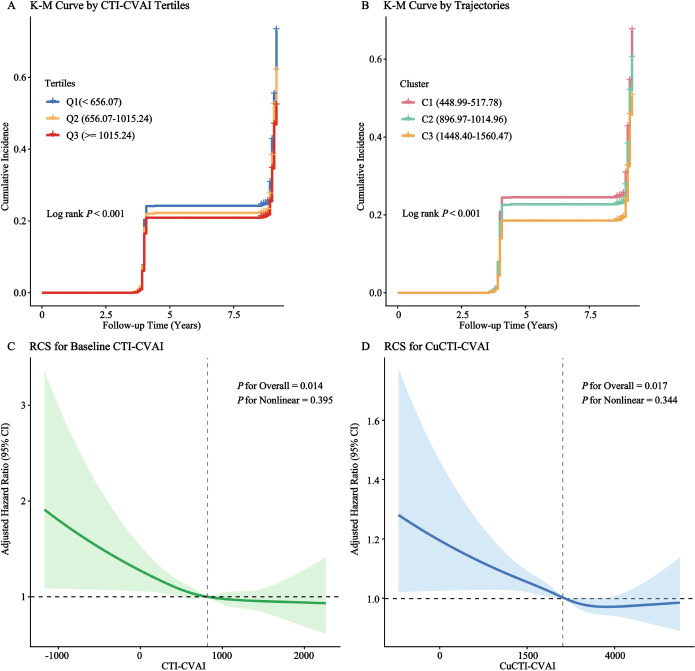
Kaplan-Meier survival curves and restricted cubic spline (RCS) analyses for incident depression according to CTI-CVAI exposures.P**(A, B)** Kaplan-Meier curves stratified by baseline tertiles and dynamic trajectories; **(C, D)** Linear dose-response relationships of baseline CTI-CVAI and cumulative CuCTI-CVAI with depression risk].

Kaplan-Meier survival curves demonstrated a progressive decrease in cumulative depression incidence across both baseline CTI-CVAI tertiles and longitudinal trajectory clusters, with the highest burden groups (Q3 and Cluster 3) exhibiting the lowest incidence (log-rank *P* < 0.001; **Figures 5A, B**).

The robust inverse association between CTI-CVAI and incident depression was confirmed using multivariable Cox proportional hazards models (**Table 2**). In the clinically-driven fully adjusted model (Model 4), each 1-SD increment in baseline CTI-CVAI was independently associated with a lower risk of incident depression (HR = 0.91, 95% CI: 0.85-0.96, *P* < 0.001). Comparable estimates were observed in the machine-learning-adjusted Model 3 (HR = 0.90, 95% CI: 0.85-0.95), demonstrating consistency across covariate-selection strategies. Compared with the lowest tertile (Q1), participants in Q3 exhibited a decreased risk in Model 4 (HR = 0.88, 95% CI: 0.76-0.98; *P* for trend < 0.001). Consistent results were observed in the longitudinal exposure analyses: relative to the low-stable group (Cluster 1), individuals in the high-increasing trajectory (Cluster 3) had a lower risk of incident depression (HR = 0.81, 95% CI: 0.69-0.97, *P* = 0.018), and those in the highest cumulative tertile (CuCTI-CVAI Q3) maintained a decreased risk compared to Q1 (HR = 0.87, 95% CI: 0.76-0.97, *P* = 0.035).

**Table 2:** Longitudinal associations of baseline, cumulative, and trajectory exposures of CTI-CVAI with the risk of incident depression.

Groups	Model1		Model2		Model3		Model4	
	HR (95%CI)	*P*	HR (95%CI)	*P*	HR (95%CI)	*P*	HR (95%CI)	*P*
CTI-CVAI Per 1SD	0.87 (0.82-0.92)	<0.001	0.89 (0.83-0.94)	<0.001	0.90 (0.85-0.95)	<0.001	0.91 (0.85-0.96)	<0.001
Q1 (< 656.07)	1.00 (Ref)		1.00 (Ref)		1.00 (Ref)		1.00 (Ref)	
Q2 (656.07-1015.24)	0.85 (0.75-0.95)	<0.001	0.86 (0.76-0.96)	0.019	0.89 (0.78-0.98)	0.022	0.92 (0.78-0.99)	0.031
Q3 (≥ 1015.24)	0.77 (0.67-0.87)	<0.001	0.81 (0.71-0.91)	0.001	0.87 (0.76-0.97)	0.018	0.88 (0.76-0.98)	0.020
*P* for trend		<0.001		<0.001		<0.001		<0.001
CTI-CVAI.Cluster								
C1 (448.99-517.78)	1.00 (Ref)		1.00 (Ref)		1.00 (Ref)		1.00 (Ref)	
C2 (896.97-1014.96)	0.85 (0.75-0.96)	0.012	0.90 (0.83-0.97)	0.013	0.93 (0.88-0.98)	0.024	0.93 (0.88-0.99)	0.028
C3 (1448.40-1560.47)	0.74 (0.64-0.87)	<0.001	0.77 (0.66-0.90)	<0.001	0.81 (0.69-0.95)	0.011	0.81 (0.69-0.97)	0.018
*P* for trend		<0.001		<0.001		<0.001		<0.001
CuCTI-CVAI Per 1SD	0.90 (0.85-0.95)	<0.001	0.91 (0.86-0.96)	<0.001	0.93 (0.89-0.98)	0.034	0.94 (0.89-0.99)	0.039
Q1 (< 2118.67)	1.00 (Ref)		1.00 (Ref)		1.00 (Ref)		1.00 (Ref)	
Q2 (2118.67-3179.46)	0.83 (0.73-0.94)	0.018	0.85 (0.75-0.97)	<0.001	0.88 (0.77-0.98)	0.033	0.90 (0.78-0.99)	0.037
Q3 (≥3179.46)	0.79 (0.69-0.90)	<0.001	0.81 (0.71-0.92)	<0.001	0.86 (0.75-0.96)	0.031	0.87 (0.76-0.97)	0.035
*P* for trend		<0.001		<0.001		<0.001		<0.001

Model1: unadjusted.

Model2: adjusted for Age, Sex, Hukou, Drinking.

Model3: adjusted for Model2 + Social isolation, Self-rated health, Cognitive score, Mental state.

Model4: adjusted for Model3 + Education, Marital, Hypertension, Diabetes, Dyslipidemia, Antidiabetic, Lipid-lowering, Antihypertensive, Nocturnal sleep time, Activities of daily living.

CuCTI-CVAI, Cumulative CTI-Chinese Visceral Adiposity Index.

Furthermore, prolonged exposure to high CTI-CVAI levels, represented by the cumulative burden (CuCTI-CVAI), was persistently associated with a lower risk. In the clinically-driven fully adjusted model, each 1-SD increment in CuCTI-CVAI was associated with a 6% lower risk of incident depression (HR = 0.94, 95% CI: 0.89-0.99), and participants in the highest cumulative tertile had a 13% lower risk than those in the lowest tertile (HR = 0.87, 95% CI: 0.76-0.97; *P* for trend < 0.001). Finally, restricted cubic spline (RCS) analyses elucidated a continuous, linear dose-response relationship, showing a stable decline in depression risk with increasing levels of both baseline CTI-CVAI (*P* for overall = 0.014, *P* for nonlinear = 0.395) and CuCTI-CVAI (*P* for overall = 0.017, *P* for nonlinear = 0.344) (**Figures 5C, D**).

### Subgroup analyses

**Figure 6:**
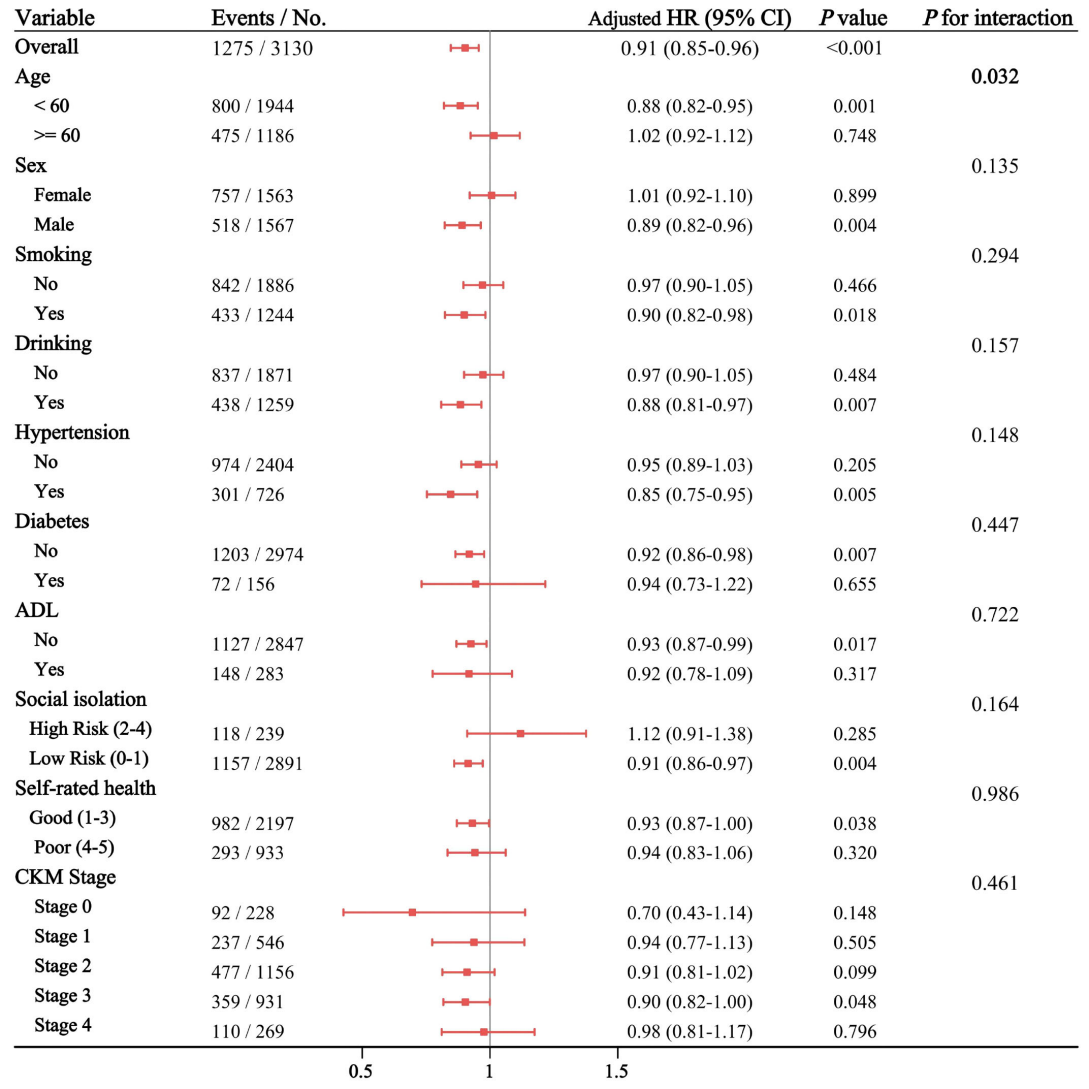
Subgroup analyses and interaction tests for the association between baseline CTI-CVAI and the risk of incident depression.

To evaluate the robustness of the associations and explore potential effect modifiers, subgroup analyses and interaction tests were performed for both baseline CTI-CVAI (**Figure 6**) and cumulative CuCTI-CVAI (**Supplementary Figure 2**). The inverse association between CTI-CVAI indices and incident depression remained largely consistent across most prespecified subgroups, including strata defined by sex, lifestyle factors (smoking and drinking), comorbidities (hypertension, diabetes), social isolation, self-rated health, and CKM stages (all *P* for interaction > 0.05).

Notably, a significant interaction was detected for age in both baseline (P for interaction = 0.032) and cumulative (*P* for interaction = 0.035) models. The inverse association of elevated CTI-CVAI against depression was highly significant among participants aged < 60 years (baseline HR: 0.88, 95% CI: 0.82-0.95; cumulative HR: 0.89, 95% CI: 0.82-0.96). However, the association was no longer observed in individuals aged ≥ 60 years (HR = 1.02 for both baseline and cumulative analyses, *P* > 0.05).

### Validation of CTI-CVAI in the independent clinical cohort

To provide directional replication of our findings, we evaluated the predictive value of CTI-CVAI in an independent real-world clinical cohort comprising 350 patients (aged ≥ 45 years) across CKM stages 0-4. During the follow-up period, 53 patients (15.1%) developed incident depression. Consistent with the primary analysis from the CHARLS cohort, the baseline CTI-CVAI levels were significantly lower in patients who developed depression compared to the non-depression controls (median: 626.76 [IQR: 436.25-758.77] vs. 889.60 [IQR: 733.13-1184.63], *P* < 0.001) (**Supplementary Table 6**).

ROC curve analysis in this validation cohort demonstrated that baseline CTI-CVAI showed moderate discriminatory ability, yielding an AUC of 0.691, which visibly outperformed the baseline clinical model (AUC: 0.655) and the CTI index (AUC: 0.672) (**Supplementary Figure 3**). Furthermore, due to the inherent limitations of retrospective EMRs in accurately capturing precise time-to-event data across all clinical visits, a multivariable logistic regression model was pragmatically employed. After comprehensively adjusting for all corresponding baseline clinical covariates aligned with Model 3, the results confirmed that a higher CTI-CVAI level remained independently associated with a lower risk. Specifically, each 1-SD increment in CTI-CVAI was associated with an 8% decreased risk of incident depression (OR = 0.92, 95% CI: 0.86-0.97, *P* < 0.001). When categorized into tertiles, participants in the highest tertile (Q3) exhibited a 21% lower risk compared to those in the lowest tertile (Q1) (OR = 0.79, 95% CI: 0.72-0.86, *P* = 0.001), demonstrating a significant dose-response trend (*P* for trend < 0.001) (**Supplementary Table 7**). These real-world data provide directional support for the inverse association of CTI-CVAI with incident depression.

## Discussion

In this nationwide prospective cohort study, we evaluated the longitudinal associations between various CTI-derived indices and the risk of incident depression among middle-aged and older Chinese adults across CKM stages 0-4. Using a multi-algorithm machine learning approach, CTI-CVAI was identified as the optimal predictor among multiple metabolic-anthropometric candidates. Our results demonstrated a consistent inverse association between CTI-CVAI and incident depression. This pattern was evident at baseline and reinforced by longitudinal dynamic trajectories and cumulative exposure analyses. Furthermore, the inverse association between elevated CTI-CVAI and depression was heterogeneous across age groups, remaining significant predominantly in individuals aged < 60 years and attenuating in older populations. Notably, these primary findings derived from the public database were directionally replicated in an independent clinical cohort, supporting the applicability of CTI-CVAI in real-world settings.

The relationship between adiposity, metabolic status, and mental health in aging populations is complex (23, 24). Recent epidemiological evidence has increasingly highlighted the substantial contribution of CKM-related components to the global disease burden (25). Within this evolving framework, advancing CKM stages have been directly associated with an elevated risk of incident depression (26). Moreover, emerging literature indicates that lipid-related metabolic indices, such as the Atherogenic Index of Plasma (AIP), are closely linked to adverse clinical outcomes in CKM populations (27). Building upon this CKM-specific context, previous epidemiological studies focusing on conventional metabolic markers, such as the TyG index or systemic inflammation (CRP), have frequently reported positive associations with depression risk, attributing this to neuroinflammation and endothelial dysfunction (28–30). However, our findings may partially echo the ‘obesity paradox’ literature, where moderate adiposity in late-life may correlate with lower depression incidence (31, 32). Unlike traditional single indicators (e.g., BMI) that cannot differentiate between fat and lean mass, or pure biochemical indices (e.g., TyG), CTI-CVAI integrates insulin resistance, systemic inflammation, and population-specific visceral adiposity (CVAI) (33–35). By capturing both the metabolic state and anthropometric reserves, CTI-CVAI provides a multifaceted reflection of an individual’s overall cardiometabolic and nutritional capacity (36–38), which may explain its moderate yet best-performing discriminatory ability among the candidate indices examined. However, given its highly composite nature—incorporating age-related body composition, lipid metabolism, insulin resistance, and systemic inflammation—its precise biological meaning is inherently complex. It is difficult to completely isolate whether the observed inverse association is driven strictly by adiposity, lipid availability, or a mixture of these components. Therefore, rather than reflecting a single, clean causal pathway, CTI-CVAI is more appropriately interpreted as a holistic proxy for integrated cardiometabolic reserves.

The inverse association between elevated CTI-CVAI and depression risk might partially align with the “nutritional reserve” hypothesis (39, 40). In late midlife, moderately elevated cardiometabolic reserves could theoretically provide a metabolic buffer against psychosocial stress or physiological wasting (41), while adequate lipid and glucose availabilities support central nervous system integrity (42, 43). However, this biological interpretation must be approached with caution. Participants in the highest CTI-CVAI group concurrently exhibited the most adverse cardiometabolic profiles, including elevated blood pressure, glucose, lipids, CRP, and more advanced CKM stages. This paradox makes a purely protective biological explanation complex. Alternative non-causal drivers must be seriously considered. The observed inverse association might be partially attributable to the healthy survivor effect, whereby individuals who can endure advanced cardiometabolic dysfunction without early psychological decline are inherently more resilient. In addition, residual confounding, survival bias, and selection bias tied to the requirement for complete baseline biomarker data could artificially inflate this correlation. It is also highly plausible that preclinical depressive symptoms, chronic stress, or sleep disturbances subtly induce weight loss and metabolic decline, creating a reverse causality loop. Consequently, a higher CTI-CVAI profile might partially reflect a state of survival resilience or the absence of preclinical disease wasting, rather than functioning solely as a direct neuroprotective factor.

Our longitudinal analyses of dynamic trajectories and cumulative burden (CuCTI-CVAI) provide insights that extend beyond cross-sectional observations. A methodological challenge in late-life depression research is reverse causality, where prodromal depression induces anorexia and unintentional weight loss prior to clinical diagnosis. By tracking CTI-CVAI over multiple waves, we demonstrated that individuals maintaining a high-increasing trajectory (Cluster 3) and possessing the highest cumulative exposure consistently exhibited the lowest depression incidence. This two-wave dose-response pattern helps mitigate the bias of prodromal weight loss to some extent, suggesting that the observed inverse association is related to the short-term maintenance of nutritional and metabolic indices. However, relying on a two-wave change pattern provides limited exposure history. This observational evidence is not robust enough to support a firm interpretation that sustained high CTI-CVAI exposure exerts a definitive protective effect against depression risk, reflecting instead an epidemiological correlation.

The age-dependent heterogeneity in the association between CTI-CVAI and depression warrants attention. The risk reduction was predominantly observed in participants aged < 60 years, whereas this association was attenuated in those aged ≥ 60 years. Because participants under 60 in this cohort represent a middle-aged demographic rather than a typical late-life depression population, the classic “obesity paradox” framework should not be overextended to this subgroup. In these middle-aged individuals, higher CTI-CVAI indices might simply reflect a different baseline metabolic capacity or shorter exposure duration to chronic metabolic damage. The divergence in older age (≥ 60 years) could speculatively be related to the competing risks of chronological aging and “inflammaging” (44–46). In advanced age, any potential physiological advantages are likely overshadowed by age-amplified systemic inflammation, advanced cerebrovascular atherosclerosis, and the accumulation of comorbidities (47–49). At this stage, the neurotoxic consequences of chronic low-grade inflammation (partially reflected by the CRP component) may neutralize the neuroendocrine benefits, attenuating the inverse association of CTI-CVAI in older populations (50–52). However, these mechanistic explanations remain speculative and require further physiological validation.

The practical implications of our findings relate primarily to epidemiological risk assessment rather than direct clinical intervention. CTI-CVAI integrates anthropometric obesity and systemic biochemical homeostasis into a single metric (53). Because its components (WC, weight, FBG, TG, and CRP) are routinely acquired, the current results support CTI-CVAI as an accessible candidate epidemiological marker for identifying individuals at higher risk of incident depression. However, given the observational nature of the data, the moderate discriminatory ability, and the methodological limitations of the replication cohort, these findings do not provide direct evidence to support personalized metabolic management or the intentional preservation of cardiometabolic reserves. Further prospective intervention studies are necessary to determine whether modifying these metabolic parameters can effectively translate into psychiatric benefits.

Despite these meaningful findings, several limitations should be acknowledged. First, excluding participants with missing data introduced selection bias. Our final analytical cohort (n=3,130) exhibited a lower prevalence of certain chronic comorbidities and less social isolation compared to the eligible-but-excluded population (n=6,626), warranting cautious generalization of our findings. Second, assessing incident depressive symptoms via screening scales (CES-D 10 and PHQ-9) rather than structured clinical interviews may capture transient psychological distress rather than persistent clinical depression. Furthermore, using different scales across cohorts weakens their strict comparability. Third, the bidirectional relationship between cardiometabolic dysfunction and depression complicates temporal inference. Even after excluding participants with clinical depression at baseline, subclinical psychological distress, chronic stress, appetite changes, or early functional decline may simultaneously alter baseline visceral adiposity, glucose metabolism, and systemic inflammation before clinical diagnosis. This complex interplay suggests that CTI-CVAI operates partially as a cumulative marker of preclinical health status rather than a strictly unidirectional predictor. Fourth, because CTI-CVAI is a highly composite metric, disentangling its specific biological drivers—whether primarily lipid reserves, insulin resistance, age-related body composition, or systemic inflammation—remains challenging. Fifth, given the observational design, residual confounding and survival bias inherent in aging CKM populations cannot be entirely excluded. Finally, although an independent clinical cohort was employed to corroborate our primary findings, this dataset was derived from a single-center retrospective hospital cohort with a small sample size. Because of inherent differences in the outcome instrument, follow-up structure, and the use of logistic rather than Cox regression due to incomplete time-to-event data, this analysis serves as a directional replication rather than a strict external validation. Further multicenter prospective studies employing competing risk models are required to validate these findings across broader socio-cultural contexts.

## Conclusion

In conclusion, CTI-CVAI is an independent predictor with moderate discriminatory ability for incident depression among middle-aged and older adults across CKM stages 0-4. Our longitudinal analyses indicate that higher baseline levels, elevated dynamic trajectories, and greater cumulative exposure to CTI-CVAI are associated with a decreased risk of incident depression, particularly in participants aged < 60 years. These findings position CTI-CVAI as a candidate epidemiological marker for risk stratification of incident depression, while the inverse association observed warrants cautious interpretation given the observational design.

## Data Availability

The datasets presented in this study can be found in online repositories. The names of the repository/repositories and accession number(s) can be found below: https://charls.pku.edu.cn/.
