# Supplementary material for: Association of CTI and its obesity-related derivatives with incident depression among middle-aged and older adults across CKM stages 0-4: a nationwide prospective cohort study and external clinical validation
Source: Front Endocrinol (Lausanne). 2026 May 20;17:1849662. doi: 10.3389/fendo.2026.1849662 (PMC13229798; doi:10.3389/fendo.2026.1849662)
Supplement: Supplementary file 1 [file DataSheet1.docx]

| **Table S1** Definitions and calculation formulas of the CTI and evaluated adiposity indices | | |
| --- | --- | --- |
| **Abbreviation** | **Full Name** | **Calculation Formula** |
| CTI | C-reactive protein-triglycerides-glucose index | 0.412 × In(CRP [mg/L]) + In(TG [mg/dL] × FPG [mg/dL]) / 2 |
| BMI | Body mass index | weight (kg) / height (m)² |
| WHtR | Waist circumference-to-height ratio | WC (cm) / height (cm) |
| BRI | Body roundness index | 364.2-365.5 ×√(1-(WC (cm) /2π)^2^ / (height (cm) /2) ^2^ ) |
| WWI | Weight-adjusted waist index | WC (cm) /√weight (kg) |
| ABSI | A body shape index | WC (m) / (BMI^2/3^× height (m)^1/2^) |
| VAI | Visceral adiposity index | For males: WC (cm) / (39.68+1.88×BMI) × TG (mmol/L) / 1.03×1.31 / HDL-C (mmol/L) |
|  |  | For females: WC (cm) / (36.58+1.89×BMI) × TG (mmol/L) / 0.81×1.52 / HDL-C (mmol/L) |
| CVAI | Chinese visceral adiposity index | For males: -267.93 + 0.68 × age + 0.03 × BMI + 4.00 × WC (cm) + 22.00 × log10 (TG) (mmol/L) -16.32 × HDL-C (mmol/L) |
|  |  | For females: -187.32 + 1.71 × age + 4.23 × BMI + 1.12 × WC (cm) + 39.76 × log10 (TG) (mmol/L) -11.66 × HDL-C (mmol/L) |

| **Table S2** Baseline characteristics of participants categorized by incident depression status | | | |
| --- | --- | --- | --- |
| **Variables** | **Non-Depression**  **(n = 1855)** | **Depression**  **(n = 1275)** | ***P*** |
| Age, years | 57.00 (51.00, 63.00) | 57.00 (51.00, 62.00) | 0.658 |
| SBP, mmHg | 126.00 (113.38, 139.00) | 125.50 (114.00, 141.50) | 0.155 |
| DBP, mmHg | 74.50 (67.00, 82.50) | 74.50 (67.50, 83.50) | 0.295 |
| Weight, kg | 61.00 (53.70, 68.50) | 58.40 (52.00, 65.75) | <0.001 |
| Height, m | 1.60 (1.54, 1.66) | 1.57 (1.52, 1.63) | <0.001 |
| WC, cm | 86.00 (79.00, 93.00) | 85.00 (78.50, 92.00) | 0.009 |
| BMI, kg/m^2^ | 23.74 (21.57, 26.17) | 23.43 (21.29, 25.89) | 0.030 |
| BUN, mg/dL | 15.01 (12.77, 17.81) | 15.01 (12.34, 17.84) | 0.643 |
| Cr, mg/dL | 0.77 (0.67, 0.89) | 0.75 (0.66, 0.87) | 0.005 |
| HbA1c, % | 5.10 (4.90, 5.40) | 5.10 (4.90, 5.40) | 0.544 |
| UA, mg/dL | 4.34 (3.63, 5.22) | 4.21 (3.50, 5.08) | 0.010 |
| TC, mg/dL | 189.82 (167.40, 213.98) | 190.59 (167.01, 216.30) | 0.318 |
| TG, mg/dL | 108.86 (77.00, 161.07) | 103.54 (73.46, 157.53) | 0.048 |
| HDL-C, mg/dL | 47.55 (39.05, 57.60) | 49.87 (40.98, 59.92) | 0.001 |
| LDL-C, mg/dL | 114.05 (93.17, 135.31) | 113.66 (92.40, 138.79) | 0.796 |
| CRP, mg/L | 1.04 (0.55, 2.04) | 0.95 (0.53, 1.94) | 0.115 |
| FPG, mg/dL | 102.96 (94.86, 113.22) | 102.06 (94.32, 112.68) | 0.098 |
| Cognitive score | 13.00 (10.25, 15.00) | 11.00 (7.00, 13.50) | <0.001 |
| Episodic memory score | 4.00 (2.50, 5.00) | 3.00 (1.00, 4.50) | <0.001 |
| Mental state score | 9.00 (7.00, 11.00) | 8.00 (5.00, 10.00) | <0.001 |
| Sex, n (%) |  |  | <0.001 |
| Male | 1049 (56.55) | 518 (40.63) |  |
| Female | 806 (43.45) | 757 (59.37) |  |
| Marital, n (%) |  |  | 0.006 |
| Married | 1686 (90.89) | 1120 (87.84) |  |
| Unmarried | 169 (9.11) | 155 (12.16) |  |
| Education, n (%) |  |  | <0.001 |
| < high school | 1590 (85.71) | 1192 (93.49) |  |
| ≥ high school | 265 (14.29) | 83 (6.51) |  |
| Hukou, n (%) |  |  | <0.001 |
| Rural | 759 (40.92) | 355 (27.84) |  |
| Urban | 1096 (59.08) | 920 (72.16) |  |
| Smoking, n (%) |  |  | <0.001 |
| No | 1044 (56.28) | 842 (66.04) |  |
| Yes | 811 (43.72) | 433 (33.96) |  |
| Drinking, n (%) |  |  | <0.001 |
| No | 1034 (55.74) | 837 (65.65) |  |
| Yes | 821 (44.26) | 438 (34.35) |  |
| Hypertension, n (%) |  |  | 0.650 |
| No | 1430 (77.09) | 974 (76.39) |  |
| Yes | 425 (22.91) | 301 (23.61) |  |
| Diabetes, n (%) |  |  | 0.158 |
| No | 1771 (95.47) | 1203 (94.35) |  |
| Yes | 84 (4.53) | 72 (5.65) |  |
| Dyslipidemia, n (%) |  |  | 0.236 |
| No | 1667 (89.87) | 1162 (91.14) |  |
| Yes | 188 (10.13) | 113 (8.86) |  |
| Antidiabetic, n(%) |  |  | 0.973 |
| No | 1803 (97.20) | 1239 (97.18) |  |
| Yes | 52 (2.80) | 36 (2.82) |  |
| Lipid-lowering, n (%) |  |  | 0.102 |
| No | 1754 (94.56) | 1222 (95.84) |  |
| Yes | 101 (5.44) | 53 (4.16) |  |
| Antihypertensive, n (%) |  |  | 0.953 |
| No | 1548 (83.45) | 1065 (83.53) |  |
| Yes | 307 (16.55) | 210 (16.47) |  |
| Nocturnal sleep time, n (%) |  |  | 0.006 |
| < 6 h | 347 (18.71) | 290 (22.75) |  |
| ≥ 6 h | 1508 (81.29) | 985 (77.25) |  |
| Activities of daily living, n (%) |  |  | <0.001 |
| No | 1720 (92.72) | 1127 (88.39) |  |
| Yes | 135 (7.28) | 148 (11.61) |  |
| Social isolation, n (%) |  |  | <0.001 |
| None | 880 (47.44) | 490 (38.43) |  |
| Mild | 854 (46.04) | 667 (52.31) |  |
| Moderate | 96 (5.18) | 101 (7.92) |  |
| High | 22 (1.19) | 15 (1.18) |  |
| Extreme | 3 (0.16) | 2 (0.16) |  |
| Self-rated health, n (%) |  |  | <0.001 |
| Excellent | 22 (1.19) | 31 (2.43) |  |
| Very good | 200 (10.78) | 239 (18.75) |  |
| Good | 993 (53.53) | 712 (55.84) |  |
| Fair | 450 (24.26) | 228 (17.88) |  |
| Poor | 190 (10.24) | 65 (5.10) |  |
| CTI | 8.69 (8.18, 9.30) | 8.62 (8.10, 9.18) | 0.025 |
| CTI-BMI | 206.47 (180.67, 239.19) | 201.18 (176.05, 235.21) | 0.003 |
| CTI-WC | 746.60 (658.19, 843.60) | 727.27 (642.32, 834.56) | 0.001 |
| CTI-WHtR | 4.68 (4.10, 5.30) | 4.64 (4.05, 5.29) | 0.368 |
| CTI-BRI | 35.25 (27.11, 45.92) | 35.78 (27.08, 46.07) | 0.859 |
| CTI-WWI | 95.92 (87.54, 104.57) | 96.35 (87.53, 105.71) | 0.640 |
| CTI-VAI | 13.00 (7.50, 24.49) | 12.88 (7.18, 24.25) | 0.441 |
| CTI-CVAI | 837.99 (583.14, 1156.52) | 789.58 (550.59, 1093.72) | 0.001 |
| CTI-ABSI | 7.15 (6.59, 7.71) | 7.12 (6.53, 7.77) | 0.481 |
| M (Q₁, Q₃) for continuous variables; n (%) for categorical variables. SBP, systolic blood pressure; DBP, diastolic blood pressure; WC, waist circumference; BMI, body mass index; BUN, blood urea nitrogen; Cr, serum creatinine; HbA1c, glycated hemoglobin; UA, uric acid; TC, total cholesterol; TG, triglycerides; HDL-C, high-density lipoprotein cholesterol; LDL-C, low-density lipoprotein cholesterol; CRP, C-reactive protein; FPG, fasting plasma glucose; CTI, C-reactive protein-triglyceride-glucose index; WHtR, waist-to-height ratio; BRI, body roundness index; WWI, weight-adjusted waist index; VAI, visceral adiposity index; CVAI, chinese visceral adiposity index; ABSI, a body shape index | | | |

| **Table S3** Comparison of baseline characteristics between the final analytical cohort and the excluded participants | | | | |
| --- | --- | --- | --- | --- |
| **Variables** | **Total**  **(n = 9756)** | **Excluded participants (n = 6626)** | **Final analytical cohort (n = 3130)** | ***P*** |
| Age, years | 58.00 (51.00, 65.00) | 58.00 (51.00, 66.00) | 57.00 (51.00, 63.00) | 0.406 |
| SBP, mmHg | 127.50 (115.00, 142.00) | 128.00 (115.50, 143.00) | 125.50 (113.50, 140.00) | 0.539 |
| DBP, mmHg | 75.00 (67.50, 83.50) | 75.50 (67.50, 84.00) | 74.50 (67.00, 83.00) | 0.105 |
| Weight, kg | 59.10 (52.30, 67.20) | 58.70 (51.90, 67.00) | 59.80 (53.00, 67.40) | 0.106 |
| Height, m | 1.59 (1.53, 1.65) | 1.59 (1.53, 1.66) | 1.59 (1.53, 1.65) | 0.188 |
| WC, cm | 85.00 (78.10, 92.20) | 85.00 (78.00, 92.00) | 85.60 (79.00, 92.50) | 0.800 |
| BMI, kg/m^2^ | 23.35 (21.09, 25.94) | 23.16 (20.89, 25.83) | 23.61 (21.43, 26.06) | 0.873 |
| BUN, mg/dL | 15.15 (12.60, 18.23) | 15.29 (12.63, 18.54) | 15.01 (12.58, 17.81) | 0.621 |
| Cr, mg/dL | 0.77 (0.67, 0.90) | 0.78 (0.67, 0.92) | 0.76 (0.66, 0.88) | 0.071 |
| HbA1c, % | 5.10 (4.90, 5.40) | 5.10 (4.80, 5.40) | 5.10 (4.90, 5.40) | 0.058 |
| UA, mg/dL | 4.38 (3.66, 5.28) | 4.48 (3.71, 5.36) | 4.30 (3.57, 5.18) | 0.173 |
| TC, mg/dL | 190.21 (166.62, 214.95) | 189.82 (166.24, 214.76) | 190.21 (167.40, 214.95) | 0.541 |
| TG, mg/dL | 106.20 (75.22, 157.53) | 106.20 (75.22, 154.88) | 107.08 (75.22, 159.30) | 0.342 |
| HDL-C, mg/dL | 48.71 (39.43, 58.76) | 48.71 (39.43, 59.15) | 48.33 (39.82, 58.76) | 0.651 |
| LDL-C, mg/dL | 114.43 (92.98, 136.47) | 114.82 (93.17, 136.86) | 113.66 (92.78, 136.47) | 0.955 |
| CRP, mg/L | 1.05 (0.55, 2.17) | 1.11 (0.57, 2.33) | 0.99 (0.54, 1.99) | 0.224 |
| FPG, mg/dL | 102.60 (94.50, 113.94) | 102.60 (94.32, 114.66) | 102.60 (94.68, 113.04) | 0.656 |
| Cognitive score | 12.50 (9.00, 15.00) | 12.50 (9.00, 15.00) | 12.50 (9.00, 14.50) | 0.461 |
| Episodic memory score | 4.00 (2.50, 5.00) | 4.00 (2.50, 5.00) | 3.50 (2.00, 5.00) | 0.327 |
| Mental state score | 9.00 (7.00, 11.00) | 9.00 (7.00, 11.00) | 9.00 (6.00, 10.00) | <0.001 |
| Male, n (%) | 5155 (52.84) | 3588 (54.15) | 1567 (50.06) | <0.001 |
| Married, n (%) | 8507 (87.20) | 5701 (86.04) | 2806 (89.65) | 0.262 |
| Education, n (%) |  |  |  | 0.057 |
| < high school | 8234 (84.40) | 5452 (82.28) | 2782 (88.88) |  |
| ≥ high school | 1522 (15.60) | 1174 (17.72) | 348 (11.12) |  |
| Rural, n (%) | 4373 (44.82) | 3259 (49.19) | 1114 (35.59) | <0.001 |
| Smoking, n (%) | 4067 (41.69) | 2823 (42.60) | 1244 (39.74) | 0.073 |
| Drinking, n (%) | 4025 (41.26) | 2766 (41.74) | 1259 (40.22) | 0.154 |
| Hypertension, n (%) | 2401 (24.61) | 1675 (25.28) | 726 (23.19) | 0.001 |
| Diabetes, n (%) | 559 (5.73) | 403 (6.08) | 156 (4.98) | <0.001 |
| Dyslipidemia, n (%) | 925 (9.48) | 624 (9.42) | 301 (9.62) | <0.001 |
| Antidiabetic, n(%) | 357 (3.66) | 269 (4.06) | 88 (2.81) | 0.002 |
| Lipid-lowering, n (%) | 430 (4.41) | 276 (4.17) | 154 (4.92) | 0.090 |
| Antihypertensive, n (%) | 1772 (18.16) | 1255 (18.94) | 517 (16.52) | 0.004 |
| Activities of daily living, n (%) | 897 (9.19) | 614 (9.27) | 283 (9.04) | <0.001 |
| Nocturnal sleep time, n (%) |  |  |  | 0.297 |
| < 6 h | 2800 (28.70) | 1347 (20.33) | 1453 (46.42) |  |
| ≥ 6 h | 6956 (71.30) | 5279 (79.67) | 1677 (53.58) |  |
| Social isolation, n (%) |  |  |  | <0.001 |
| None | 4117 (42.20) | 2747 (41.46) | 1370 (43.77) |  |
| Mild | 4591 (47.06) | 3070 (46.33) | 1521 (48.59) |  |
| Moderate | 815 (8.35) | 618 (9.33) | 197 (6.29) |  |
| High | 203 (2.08) | 166 (2.51) | 37 (1.18) |  |
| Extreme | 30 (0.31) | 25 (0.38) | 5 (0.16) |  |
| Self-rated health, n (%) |  |  |  | 0.140 |
| Excellent | 168 (1.72) | 115 (1.74) | 53 (1.69) |  |
| Very good | 1436 (14.72) | 997 (15.05) | 439 (14.03) |  |
| Good | 5147 (52.76) | 3442 (51.95) | 1705 (54.47) |  |
| Fair | 2189 (22.44) | 1511 (22.80) | 678 (21.66) |  |
| Poor | 810 (8.30) | 555 (8.38) | 255 (8.15) |  |
| CKM stage, n(%) |  |  |  | <0.001 |
| Stage 0 | 744 (7.63) | 516 (7.79) | 228 (7.28) |  |
| Stage 1 | 1461 (14.98) | 915 (13.81) | 546 (17.44) |  |
| Stage 2 | 3019 (30.95) | 1863 (28.12) | 1156 (36.93) |  |
| Stage 3 | 2506 (25.69) | 1575 (23.77) | 931 (29.74) |  |
| Stage 4 | 1036 (10.62) | 767 (11.58) | 269 (8.59) |  |
| SBP, systolic blood pressure; DBP, diastolic blood pressure; WC, waist circumference; BMI, body mass index; BUN, blood urea nitrogen; Cr, serum creatinine; HbA1c, glycated hemoglobin; UA, uric acid; TC, total cholesterol; TG, triglycerides; HDL-C, high-density lipoprotein cholesterol; LDL-C, low-density lipoprotein cholesterol; CRP, C-reactive protein; FPG, fasting plasma glucose; CKM, cardiovascular kidney-metabolic syndrome | | | | |

| **Table S4** Incremental predictive value of CTI-CVAI for incident depression beyond the baseline clinical model | | | | | | |
| --- | --- | --- | --- | --- | --- | --- |
| **Model** | **NRI** | | **IDI** | | **C-statistics** | |
|  | **Index (95% CI)** | ***P*** | **Index (95% CI)** | ***P*** | **Index (95% CI)** | ***P*** |
| Baseline model |  | Ref |  | Ref | 0.674 (0.655-0.693) | <0.001 |
| + CTI-CVAI | 0.126 (0.072-0.181) | <0.001 | 0.005 (0.003-0.007) | <0.001 | 0.705 (0.686-0.723) | <0.001 |
| The baseline clinical model was adjusted for age, sex, hukou, drinking, social isolation, self-rated health, cognitive score, and mental state. NRI, net reclassification improvement; IDI, integrated discrimination improvement; CI, confidence interval | | | | | | |

| **Table S5** Baseline characteristics of participants grouped by CTI-CVAI dynamic trajectory clusters | | | | |
| --- | --- | --- | --- | --- |
| **Variables** | **C1 (n = 1092)** | **C2 (n = 1359)** | **C3 (n = 679)** | ***P*** |
| Depression, n(%) | 475 (43.50) | 557 (40.99) | 243 (35.79) | 0.006 |
| Age, years | 56.00 (50.00,61.00) | 57.00 (51.00,62.00) | 59.00 (54.00,64.50) | <0.001 |
| SBP, mmHg | 119.50 (110.00,132.50) | 126.00 (113.50,140.00) | 134.50 (122.00,148.50) | <0.001 |
| DBP, mmHg | 71.50 (64.50,79.50) | 75.00 (68.00,83.50) | 79.50 (71.00,87.12) | <0.001 |
| Weight, kg | 53.50 (48.68,58.40) | 61.20 (55.20,66.60) | 71.70 (65.30,78.20) | <0.001 |
| Height, m | 1.59 (1.53,1.65) | 1.58 (1.52,1.65) | 1.60 (1.54,1.67) | <0.001 |
| WC, cm | 77.20 (73.00,81.10) | 87.00 (83.00,91.00) | 97.40 (93.00,102.00) | <0.001 |
| BMI, kg/m2 | 21.09 (19.58,22.60) | 24.11 (22.63,25.78) | 27.66 (25.72,29.66) | <0.001 |
| BUN, mg/dL | 15.11 (12.60,18.18) | 14.85 (12.38,17.62) | 15.13 (12.88,17.67) | 0.020 |
| Cr, mg/dL | 0.76 (0.66,0.87) | 0.75 (0.64,0.87) | 0.78 (0.69,0.92) | <0.001 |
| HbA1c, % | 5.10 (4.80,5.30) | 5.10 (4.90,5.40) | 5.30 (5.00,5.60) | <0.001 |
| UA, mg/dL | 4.10 (3.41,4.92) | 4.23 (3.55,5.04) | 4.80 (4.03,5.69) | <0.001 |
| TC, mg/dL | 181.90 (160.83,208.09) | 192.14 (169.33,214.95) | 197.94 (174.36,225.00) | <0.001 |
| TG, mg/dL | 77.00 (60.18,103.77) | 111.51 (83.19,154.88) | 179.65 (128.32,271.70) | <0.001 |
| HDL-C, mg/dL | 56.44 (47.17,66.88) | 47.55 (40.59,55.67) | 38.66 (32.09,46.39) | <0.001 |
| LDL-C, mg/dL | 107.86 (88.14,129.51) | 117.53 (97.42,141.88) | 116.95 (92.40,139.56) | <0.001 |
| CRP, mg/L | 0.66 (0.40,1.27) | 1.05 (0.60,2.06) | 1.58 (0.92,2.98) | <0.001 |
| FPG, mg/dL | 99.54 (92.16,107.32) | 102.60 (95.40,111.69) | 109.80 (99.72,126.72) | <0.001 |
| Cognitive score | 12.00 (9.00,14.50) | 12.50 (9.00,15.00) | 12.50 (9.00,15.00) | 0.007 |
| Episodic memory score | 3.50 (2.00,5.00) | 3.50 (2.00,5.00) | 3.50 (2.00,4.50) | 0.107 |
| Mental state score | 9.00 (6.00,10.00) | 9.00 (6.00,10.00) | 9.00 (7.00,10.50) | 0.009 |
| Sex, n (%) |  |  |  | <0.001 |
| Male | 647 (59.25) | 573 (42.16) | 347 (51.10) |  |
| Female | 445 (40.75) | 786 (57.84) | 332 (48.90) |  |
| Marital, n (%) |  |  |  | 0.403 |
| Married | 985 (90.20) | 1207 (88.82) | 614 (90.43) |  |
| Unmarried | 107 (9.80) | 152 (11.18) | 65 (9.57) |  |
| Education, n (%) |  |  |  | 0.324 |
| < high school | 983 (90.02) | 1198 (88.15) | 601 (88.51) |  |
| ≥ high school | 109 (9.98) | 161 (11.85) | 78 (11.49) |  |
| Hukou, n (%) |  |  |  | <0.001 |
| Rural | 308 (28.21) | 508 (37.38) | 298 (43.89) |  |
| Urban | 784 (71.79) | 851 (62.62) | 381 (56.11) |  |
| Smoking, n (%) |  |  |  | <0.001 |
| No | 570 (52.20) | 905 (66.59) | 411 (60.53) |  |
| Yes | 522 (47.80) | 454 (33.41) | 268 (39.47) |  |
| Drinking, n (%) |  |  |  | <0.001 |
| No | 600 (54.95) | 882 (64.90) | 389 (57.29) |  |
| Yes | 492 (45.05) | 477 (35.10) | 290 (42.71) |  |
| Hypertension, n (%) |  |  |  | <0.001 |
| No | 983 (90.02) | 1039 (76.45) | 382 (56.26) |  |
| Yes | 109 (9.98) | 320 (23.55) | 297 (43.74) |  |
| Diabetes, n (%) |  |  |  | <0.001 |
| No | 1073 (98.26) | 1288 (94.78) | 613 (90.28) |  |
| Yes | 19 (1.74) | 71 (5.22) | 66 (9.72) |  |
| Dyslipidemia, n (%) |  |  |  | <0.001 |
| No | 1049 (96.06) | 1245 (91.61) | 535 (78.79) |  |
| Yes | 43 (3.94) | 114 (8.39) | 144 (21.21) |  |
| Antidiabetic, n(%) |  |  |  | <0.001 |
| No | 1080 (98.90) | 1328 (97.72) | 634 (93.37) |  |
| Yes | 12 (1.10) | 31 (2.28) | 45 (6.63) |  |
| Lipid-lowering, n (%) |  |  |  | <0.001 |
| No | 1079 (98.81) | 1295 (95.29) | 602 (88.66) |  |
| Yes | 13 (1.19) | 64 (4.71) | 77 (11.34) |  |
| Antihypertensive, n (%) |  |  |  | <0.001 |
| No | 1024 (93.77) | 1142 (84.03) | 447 (65.83) |  |
| Yes | 68 (6.23) | 217 (15.97) | 232 (34.17) |  |
| Nocturnal sleep time, n (%) |  |  |  | 0.254 |
| < 6 h | 240 (21.98) | 265 (19.50) | 132 (19.44) |  |
| ≥ 6 h | 852 (78.02) | 1094 (80.50) | 547 (80.56) |  |
| Activities of daily living, n (%) |  |  |  | <0.001 |
| No | 1011 (92.58) | 1243 (91.46) | 593 (87.33) |  |
| Yes | 81 (7.42) | 116 (8.54) | 86 (12.67) |  |
| Social isolation, n (%) |  |  |  | 0.005 |
| None | 430 (39.38) | 611 (44.96) | 329 (48.45) |  |
| Mild | 566 (51.83) | 648 (47.68) | 307 (45.21) |  |
| Moderate | 84 (7.69) | 79 (5.81) | 34 (5.01) |  |
| High | 9 (0.82) | 20 (1.47) | 8 (1.18) |  |
| Extreme | 3 (0.27) | 1 (0.07) | 1 (0.15) |  |
| Self-rated health, n (%) |  |  |  | 0.298 |
| Excellent | 17 (1.56) | 24 (1.77) | 12 (1.77) |  |
| Very good | 155 (14.19) | 188 (13.83) | 96 (14.14) |  |
| Good | 611 (55.95) | 746 (54.89) | 348 (51.25) |  |
| Fair | 238 (21.79) | 285 (20.97) | 155 (22.83) |  |
| Poor | 71 (6.50) | 116 (8.54) | 68 (10.01) |  |
| CKM stage, n(%) |  |  |  | <0.001 |
| Stage 0 | 200 (18.32) | 27 (1.99) | 1 (0.15) |  |
| Stage 1 | 274 (25.09) | 256 (18.84) | 16 (2.36) |  |
| Stage 2 | 316 (28.94) | 597 (43.93) | 243 (35.79) |  |
| Stage 3 | 246 (22.53) | 374 (27.52) | 311 (45.80) |  |
| Stage 4 | 56 (5.13) | 105 (7.73) | 108 (15.91) |  |
| SBP, systolic blood pressure; DBP, diastolic blood pressure; WC, waist circumference; BMI, body mass index; BUN, blood urea nitrogen; Cr, serum creatinine; HbA1c, glycated hemoglobin; UA, uric acid; TC, total cholesterol; TG, triglycerides; HDL-C, high-density lipoprotein cholesterol; LDL-C, low-density lipoprotein cholesterol; CRP, C-reactive protein; FPG, fasting plasma glucose; CKM, cardiovascular kidney-metabolic syndrome | | | | |

| **Table S6** Baseline characteristics of participants categorized by incident depression status in the independent clinical validation cohort | | | | |
| --- | --- | --- | --- | --- |
| **Variables** | **Total**  **(n = 350)** | **Non-Depression**  **(n = 297)** | **Depression**  **(n = 53)** | ***P*** |
| Age, years | 56.00 (51.00, 61.00) | 56.00 (51.00, 61.00) | 56.00 (50.00, 58.00) | 0.224 |
| WC, cm | 84.00 (77.62, 91.20) | 84.00 (78.00, 91.28) | 83.70 (73.85, 90.25) | 0.016 |
| BMI, kg/m^2^ | 23.53 (21.41, 26.05) | 24.54 (22.06, 28.29) | 23.36 (21.31, 25.79) | 0.010 |
| TG, mg/dL | 114.17 (81.42, 171.47) | 119.65 (75.62, 187.13) | 110.62 (80.76, 167.71) | 0.027 |
| HDL-C, mg/dL | 48.71 (40.98, 59.05) | 47.13 (40.37, 55.15) | 48.90 (40.69, 59.83) | 0.008 |
| CRP, mg/L | 0.99 (0.55, 1.99) | 1.22 (0.56, 2.63) | 0.92 (0.55, 1.89) | 0.292 |
| FPG, mg/dL | 101.88 (94.50, 110.43) | 101.66 (95.10, 113.81) | 101.16 (93.82, 108.68) | 0.068 |
| CTI | 8.73 (8.19, 9.33) | 8.92 (8.28, 9.64) | 8.66 (8.17, 9.23) | 0.039 |
| CTI-CVAI | 843.50 (704.85, 1100.88) | 889.60 (733.13, 1184.63) | 626.76 (436.25, 758.77) | <0.001 |
| Cognitive score | 12.50 (9.00, 15.00) | 13.00 (9.50, 15.00) | 11.00 (7.00, 13.50) | <0.001 |
| Mental state score | 9.00 (6.00, 10.00) | 9.00 (7.00, 11.00) | 7.00 (4.00, 9.00) | <0.001 |
| Sex, n (%) |  |  |  | 0.036 |
| Male | 172 (49.14) | 153 (51.52) | 19 (35.85) |  |
| Female | 178 (50.86) | 144 (48.48) | 34 (64.15) |  |
| Hukou, n (%) |  |  |  | 0.013 |
| Rural | 118 (33.71) | 108 (36.36) | 10 (18.87) |  |
| Urban | 232 (66.29) | 189 (63.64) | 43 (81.13) |  |
| Drinking, n (%) |  |  |  | 0.028 |
| No | 217 (62.00) | 177 (59.60) | 40 (75.47) |  |
| Yes | 133 (38.00) | 120 (40.40) | 13 (24.53) |  |
| Social isolation, n (%) |  |  |  | 0.022 |
| None | 136 (38.86) | 124 (41.75) | 12 (22.64) |  |
| Mild | 189 (54.00) | 154 (51.85) | 35 (66.04) |  |
| Moderate | 17 (4.86) | 14 (4.71) | 3 (5.66) |  |
| High | 6 (1.71) | 4 (1.35) | 2 (3.77) |  |
| Extreme | 2 (0.57) | 1 (0.34) | 1 (1.89) |  |
| Self-rated health, n (%) |  |  |  | <0.001 |
| Excellent | 13 (3.71) | 6 (2.02) | 7 (13.21) |  |
| Very good | 51 (14.57) | 40 (13.47) | 11 (20.75) |  |
| Good | 193 (55.14) | 168 (56.57) | 25 (47.17) |  |
| Fair | 72 (20.57) | 63 (21.21) | 9 (16.98) |  |
| Poor | 21 (6.00) | 20 (6.73) | 1 (1.89) |  |
| WC, waist circumference; BMI, body mass index; TG, triglycerides; HDL-C, high-density lipoprotein cholesterol; CRP, C-reactive protein; FPG, fasting plasma glucose; CTI, C-reactive protein-triglyceride-glucose index; CVAI, chinese visceral adiposity index | | | | |

| **Table S7** Association of baseline CTI-CVAI with the risk of incident depression in the independent clinical validation cohort | | | | | | | |
| --- | --- | --- | --- | --- | --- | --- | --- |
| **Variables** | **Groups** | **Model1** | | **Model2** | | **Model3** | |
|  |  | **OR (95%CI)** | ***P*** | **OR (95%CI)** | ***P*** | **OR (95%CI)** | ***P*** |
| **CTI-CVAI** | Per 1SD | 0.86 (0.80-0.91) | <0.001 | 0.88 (0.82-0.94) | <0.001 | 0.92 (0.86-0.97) | <0.001 |
|  | Q1 (< 736.56) | 1.00 (Ref) |  | 1.00 (Ref) |  | 1.00 (Ref) |  |
|  | Q2 (736.56-994.26) | 0.82 (0.73-0.92) | <0.001 | 0.84 (0.75-0.94) | 0.006 | 0.88 (0.81-0.95) | 0.013 |
|  | Q3 (≥ 994.26) | 0.73 (0.65-0.81) | <0.001 | 0.76 (0.70-0.83) | 0.001 | 0.79 (0.72-0.86) | 0.001 |
| *P* for trend |  |  | <0.001 |  | <0.001 |  | <0.001 |
| Model1: unadjusted  Model2: adjusted for Age, Sex, Hukou, Drinking  Model3: adjusted for Model2 + Social isolation, Self-rated health, Cognitive score, Mental state | | | | | | | |

| 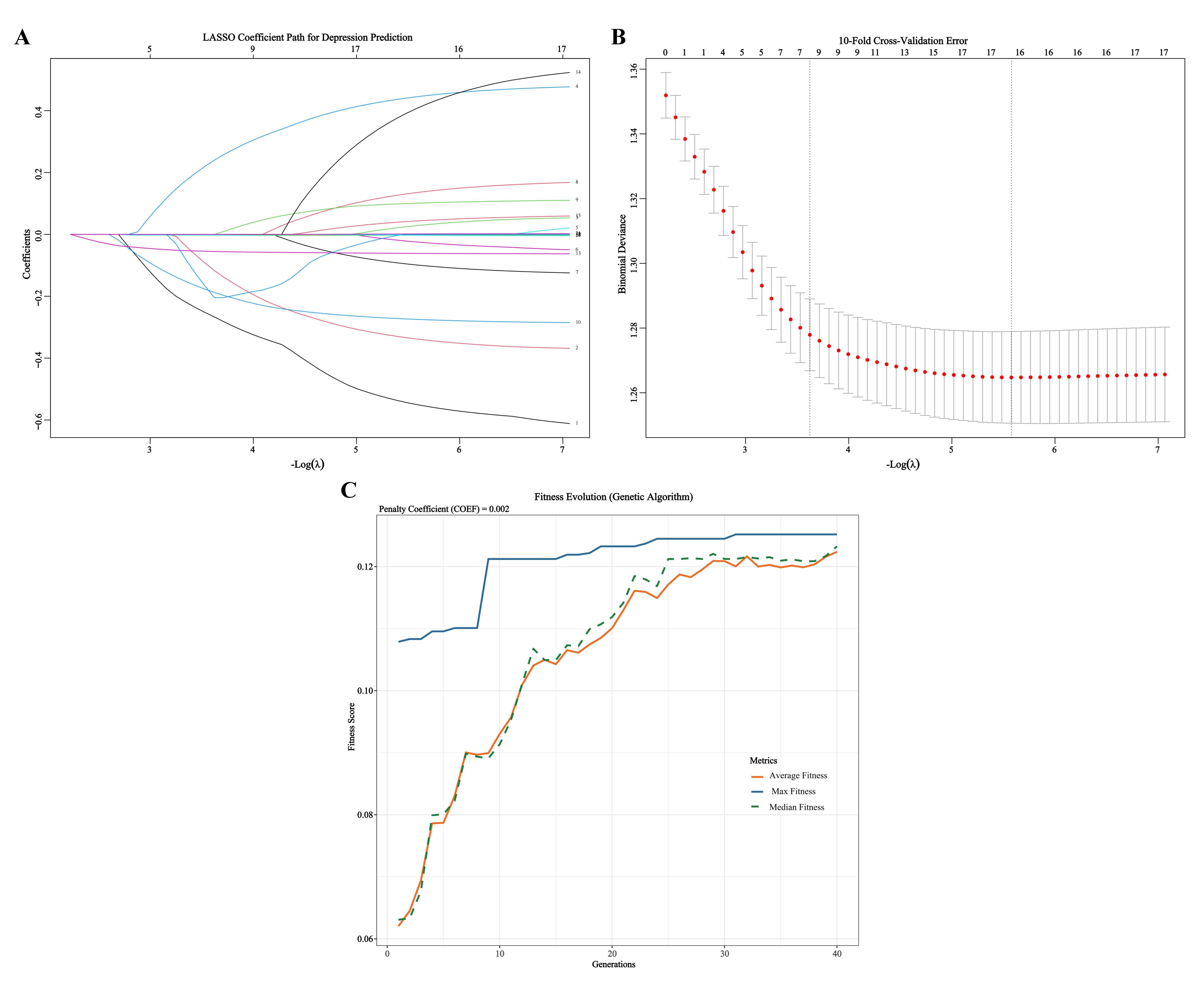 |
| --- |
| **Fig. S1** Detailed feature selection processes of the LASSO regression and Genetic Algorithm (GA). (A: Cross-validation curve for tuning parameter selection in the LASSO model; B: Coefficient shrinkage paths of the evaluated features in the LASSO model; C: Iterative fitness curve illustrating the optimization process of the GA) |

| 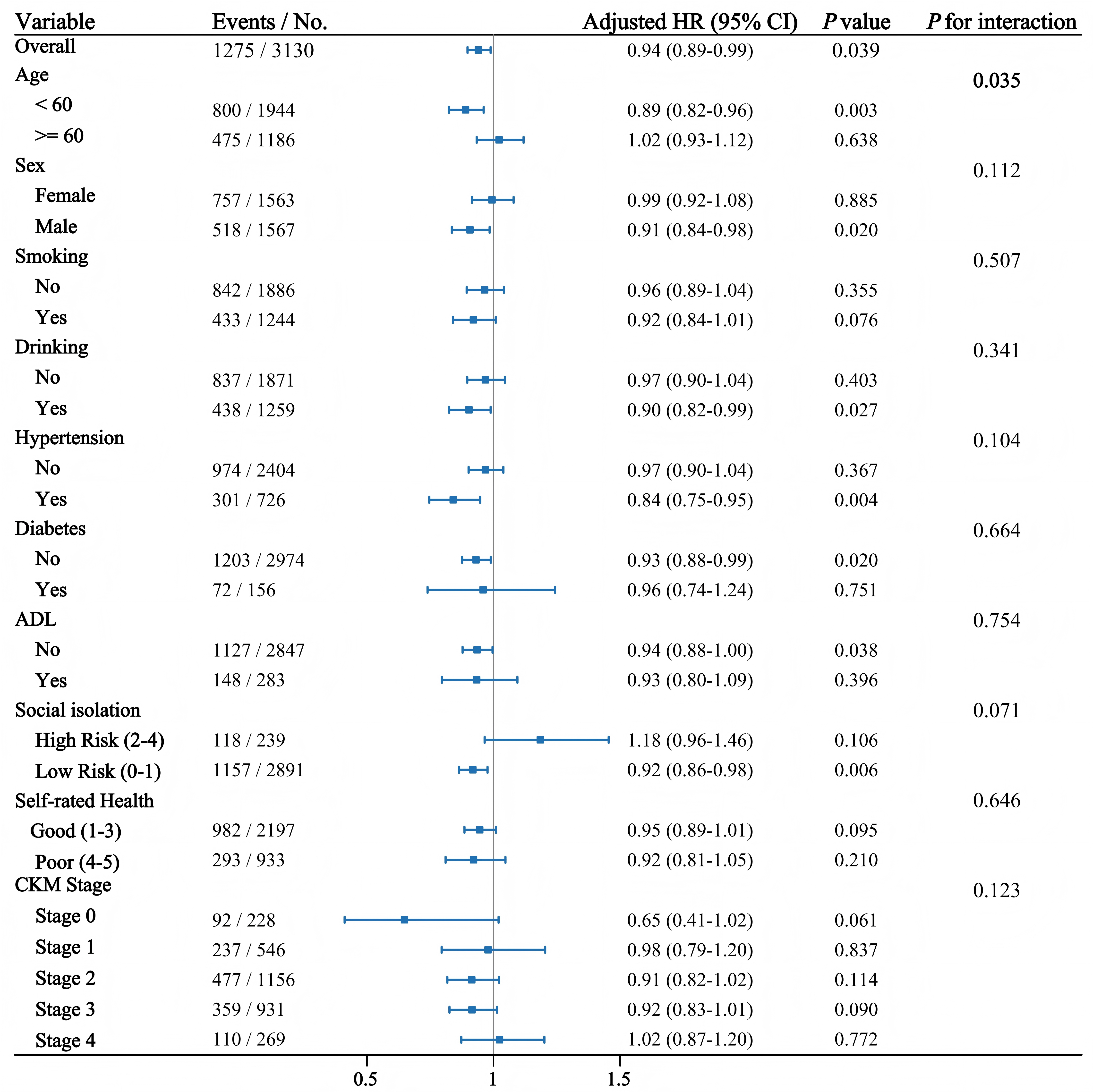 |
| --- |
| **Fig. S2** Subgroup analyses and interaction tests for the association between CuCTI-CVAI and the risk of incident depression |

|  |
| --- |
| **Fig. S3** ROC curves of CTI-CVAI, CTI, and the baseline clinical model for predicting incident depression in the independent clinical validation cohort |
